# A scalable assembly-free variable selection algorithm for biomarker discovery from metagenomes

**DOI:** 10.1186/s12859-016-1186-3

**Published:** 2016-08-19

**Authors:** Anestis Gkanogiannis, Stéphane Gazut, Marcel Salanoubat, Sawsan Kanj, Thomas Brüls

**Affiliations:** 1 Commissariat à l’Energie Atomique et aux Energies Alternatives, Direction de la Recherche Fondamentale, Institut de Génomique, Genoscope, Evry, Essonne 91057 France; 2 UMR 8030 – Génomique Métabolique, Centre National de la Recherche Scientifique, Evry, Essonne 91057 France; 3 Université d’Evry-Val-d’Essonne & Université Paris-Saclay, Evry, Essonne 91000 France; 4 Commissariat à l’Energie Atomique et aux Energies Alternatives, Direction de la Recherche Technologique, CEA-Tech, LIST, Laboratoire d’Analyse de Données et Intelligence des Systèmes, 91191 Gif-sur-Yvette, France

**Keywords:** Metagenomics, Binning, Unsupervised learning, Environmental genomics, Microbiome, Sequence clustering

## Abstract

**Background:**

Metagenomics holds great promises for deepening our knowledge of key bacterial driven processes, but metagenome assembly remains problematic, typically resulting in representation biases and discarding significant amounts of non-redundant sequence information. In order to alleviate constraints assembly can impose on downstream analyses, and/or to increase the fraction of raw reads assembled via targeted assemblies relying on pre-assembly binning steps, we developed a set of binning modules and evaluated their combination in a new “assembly-free” binning protocol.

**Results:**

We describe a scalable multi-tiered binning algorithm that combines frequency and compositional features to cluster unassembled reads, and demonstrate i) significant runtime performance gains of the developed modules against state of the art software, obtained through parallelization and the efficient use of large lock-free concurrent hash maps, ii) its relevance for clustering unassembled reads from high complexity (e.g., harboring 700 distinct genomes) samples, iii) its relevance to experimental setups involving multiple samples, through a use case consisting in the “de novo” identification of sequences from a target genome (e.g., a pathogenic strain) segregating at low levels in a cohort of 50 complex microbiomes (harboring 100 distinct genomes each), in the background of closely related strains and the absence of reference genomes, iv) its ability to correctly identify clusters of sequences from the *E. coli O104:H4* genome as the most strongly correlated to the infection status in 53 microbiomes sampled from the 2011 STEC outbreak in Germany, and to accurately cluster contigs of this pathogenic strain from a cross-assembly of these 53 microbiomes.

**Conclusions:**

We present a set of sequence clustering (“binning”) modules and their application to biomarker (e.g., genomes of pathogenic organisms) discovery from large synthetic and real metagenomics datasets. Initially designed for the “assembly-free” analysis of individual metagenomic samples, we demonstrate their extension to setups involving multiple samples via the usage of the “alignment-free” d_2_S statistic to relate clusters across samples, and illustrate how the clustering modules can otherwise be leveraged for *de novo* “pre-assembly” tasks by segregating sequences into biologically meaningful partitions.

**Electronic supplementary material:**

The online version of this article (doi:10.1186/s12859-016-1186-3) contains supplementary material, which is available to authorized users.

## Background

Since the early pioneering studies that were mostly exploratory in nature, metagenomics is now being applied in more quantitative settings [1]. However, many analyses typically start with metagenome assembly which, despite significant efforts and noticeable improvements [2, 3], remains a challenging task. Representational biases induced by the varying abundance levels of the different genomes making up a community genome are a recognized problem [4]. For example, Table 1 illustrates the breakdown of a global metagenome assembly into different abundance classes defined by clustering the unassembled (raw) reads with the coverage-based binning module presented hereafter. The mapping of the binned reads on the metagenome assembly reveals a strong bias toward 30x coverage reads, while both more abundant and less abundant reads (and hence the genomes these reads originate from) are significantly under-represented in the assembly. To increase the amount of sequence information extractable from raw datasets, we developed and evaluated an “assembly-free” analytical pipeline combining frequency (coverage) and compositional features to partition unassembled sequences in a fully unsupervised way, and evaluated the accuracy and usefulness of the resulting sequence sets in several ways (see Methods): i) via extensive benchmarking against state of the art methods, using synthetic metagenomic datasets made of up to 700 distinct genomes under realistic abundance and sequence error models, ii) by evaluating the newly developed clustering modules on real metagenomics datasets derived from both environmental bioremediation projects and human microbiomes, iii) by correlating the output clusters, treated as variables, with metadata labels (i.e., health status) for a virtual cohort of 50 microbiomes of 100 organisms each, originating from 40 healthy and 10 sick individuals, the latter being spiked at low levels with a mock “pathogenic” strain, iv) by complementing this virtual cohort experiment with an analysis of a real-world STEC O104:H4 outbreak dataset [5] based on our compositional binning module, in order to evaluate its ability to identify and partition sequences from the disease-causing genome.

**Table 1:** Coverage biases in metagenome assemblies

Abundance class (Bin #)	Bin abundance level	% Binned reads in assembly	Estimated number of genomes in bin
Bin I	320	2.8 %	> = 3
Bin II	180	2.9 %	> = 1
Bin III	90	12.6 %	> = 2
Bin IV	30	54.0 %	> = 6
Bin V	9	0.5 %	> = 3

Unassembled (raw) reads derived from a xenobiotic degrading bacterial consortium (Chaussonerie et al. 2016 under review) were segregated by the AB-Cl module (k-mer size = 25) into 5 abundance classes (bins). Mapping of reads from individual bins on the metagenome assembly based on all the raw reads reveals a significant under-representation of abundance classes I, II, III and V

## Methods

### Background

Two types of sequence features are commonly used by “assembly-free” methods for sequence comparisons. On one hand, compositional signatures are based on the distribution of short k-mers (e.g., of length 4 to 8); they can capture genome and clade specific signals [6] and form the core of “alignment-free” methods. However, the robustness of the compositional signal is dependent on sequence length, which explains why many binning methods depending on compositional signatures, or making a joint usage of these together with coverage-based information [5, 7], rely on assembled contigs as input data. On the contrary, abundance based methods rely on long –hence supposedly unique- k-mers (e.g. 20-mers or longer) to capture genome coverage information, and are not affected by this limitation; they can thus be used to segregate very short reads (e.g., 50 bp) into different abundance classes [8]. The possibility to partition reads on the basis of a frequency criteria is relevant for many ecosystems and environments, including the human gut where variation in cell numbers between species can span four orders of magnitude [9]. The observation that the performance of many centroid-based clustering algorithms degrades when dealing with steep abundance distributions further argues for the relevance of an abundance normalizing pre-processing step.

### Generation of synthetic datasets

#### Benchmark datasets

In order to evaluate our abundance and composition-based binning modules, we generated synthetic datasets of increasing complexity, ranging from 5 to 700 different genotypes, at 2 different read lengths (150 and 600 bp) and under different abundance distributions for the sampled organisms. We assumed that species levels follow a power-law distribution, i.e., the abundance of the x^th^ species is defined by: *A*(*x*) = *βx*^− *α*^, where α is the power-law parameter and β a normalization parameter such that ∑_*x* ∈ *S*_*A*(*x*) = 1, and S designates the set of organisms. The mean coverage of the datasets was fixed to 10X and 1X for two distinct series. Two types of datasets, embodying different abundance patterns were created: the first with fixed parameter α = 0.2, and the second where this parameter was a function of the dataset’s complexity (richness) |S|. For datasets of the latter type, α is fixed in a way such that the abundance of the most abundant species in the set is four orders of magnitude greater than that of the least abundant species. Figure 2 shows organism abundances (in logarithmic scale) for datasets of different richness.

#### Datasets for the biomarker discovery use case

For the biomarker discovery use case, we generated a dataset consisting of reads from 50 different samples, e.g., 50 stool microbiomes originating from 50 different individuals. Each microbiome contains sequences sampled from 100 different bacteria randomly selected from a pool of 700 distinct bacterial genomes (Additional file 1: Table S2). The average coverage for each sample is fixed to 10X (note that this means that the bulk of species populating the long tail of the abundance distribution are sampled well below this level) and read length is set to 400 nucleotides, a length that can be achieved nowadays on several Illumina platforms (e.g., using overlapping paired-end sequencing schemes on the MiSeq platform or HiSeq 2500 v2 reagents for rapid run mode). A power-law with parameter α = 1.0 was used to model the within sample abundance distribution. In order to probe whether our analytical pipeline could identify “de novo” pathogen related sequences from the samples, we randomly picked one bacterial genome, marked it as disease causing (“pathogen”), and removed it from the pool of genomes before sampling the reads. We then created 40 “healthy” microbiomes by sampling 7.5 M reads for each of them from 100 random genomes drawn from the reservoir of reference genomes, as described earlier. The last 10 microbiomes (i.e., those derived from the 10 “sick” individuals) were then created by sampling 7.425 M reads from 99 genomes from the reservoir according to the same abundance distribution, the remaining 75 K reads (1 % of the total reads) being uniformly sampled from the genome marked as pathogen.

In addition to the genome from the “pathogen” strain (arbitrarily chosen as *Haemophilus parasuis ZJ0906* in this study), the synthetic microbiomes also included the genome of a closely related strain (*Haemophilus parasuis SH0165*), considered “harmless” in our experiments, as well as two further *Haemophilus* genomes: *Haemophilus parainfluenzae T3T1* and *Haemophilus somnus 2336* (see Additional file 1: Table S2).

For all the synthetic datasets, the read generation process was performed using the mason software [10]. This program can sample reads of the Illumina type from reference genomes under various abundance distributions, while also inserting position specific sequence modifications according to empirically calibrated and sequencing platform dependent error models. Figure 3 shows abundance levels of the 700 bacterial genomes sampled across the 50 microbiomes.

### Clustering evaluation

#### Class definitions

To evaluate the performance of the various clustering modules on the benchmark datasets, we compared class memberships of elements (reads) in each dataset to the memberships induced by the clustering.

Class membership of elements is trivial to define for datasets used in the composition-based clustering experiments, simply consisting in the genomes the reads were sampled from, and the cardinality of the class set equating the sample’s richness.

Reference classes are more tricky to define in the coverage-based binning experiments, as genomes from organisms of similar abundance need to be evaluated together. To achieve this, we devised a greedy heuristic relying on the assumption that the abundance difference between any two genomes being separable by abundance-based methods has to be at least two-fold [8]. The heuristic first sorts the relative abundances of each organism according to the power-law distribution, assuming a four order of magnitude span in abundance levels. Then, starting with the most abundant species, it iteratively adds species into groups that are broken whenever the cumulative relative abundance of a group is more than twice that of its nearest less abundant neighbor. Abundance classes generated in this way from the 700 organisms metagenome are shown with different color coding in Additional file 2: Figure S1.

#### Evaluation metrics

In order to evaluate the quality of the clustering produced by the various modules, we use the V-measure [11], an external entropy-based clustering evaluation metric which evaluates clustering solutions independently of the clustering algorithm or dataset size, and measures how successfully the clustering satisfies the criteria of homogeneity and completeness (V-measure is the harmonic mean of these two values). A perfect clustering satisfies the homogeneity criteria when each of the clusters is made up of reads that belong to a single class. On the other hand, a perfect clustering satisfies the completeness criteria when, for each class, all reads of the class are assigned to a single cluster. The known read to genome memberships provide the ground truth for these evaluations.

### Algorithm

#### Rationale

Our binning algorithm consists in three distinct modules: an initial coverage-based binning module and two composition-based ones, with the first two modules performing intra-sample sequence partitioning, while the third module carries out inter-sample comparisons to deal with experimental configurations involving multiple samples.

The abundance module starts with the computation of a sample’s k-mer spectrum (or frequency histogram, summarizing the occurrence patterns of distinct long k-mers as a function of their abundance level), and fits the parameters of a mixture model of Poisson distributions iteratively via an Expectation-Maximization (EM) process to partition the observed k-mers into distinct abundance classes. In a later stage, reads are segregated into abundance clusters based on their content in long k-mers.

The sequences from each distinct coverage-based cluster outputted by the first module are next separately processed by a composition-based module performing a k-means clustering using Spearman’s footrule distance.

The first two modules thus operate at the level of a single sample. To address the need for inter-sample comparisons and leverage the information available in such experimental setups, a third compositional module was developed in order to map -across samples- the sequence clusters generated by the first two modules at the sample level. This is accomplished by computing a similarity matrix between the second level (2L) clusters based on the d_2_S “alignment-free” statistic [12], followed by hierarchical clustering and dynamic cutting [13] of the resulting dendrogram, yielding to the definition of third level (3L) clusters.

We chose to thoroughly evaluate this design using unassembled reads of a length (150 nucleotides for coverage-based experiments and 400 nucleotides for the composition-based ones) that can be achieved on contemporary sequencing platforms. The different modules are next described in more details.

#### Abundance-based clustering

The main steps involve: a) long k-mer counting and k-mer spectrum construction, b) identification of a mixture model of Poisson distributions from the k-mer spectrum, c) reads to cluster assignments. We further detail each of these steps in the following paragraphs.

##### k-mer counting step

**Fig. 1:**
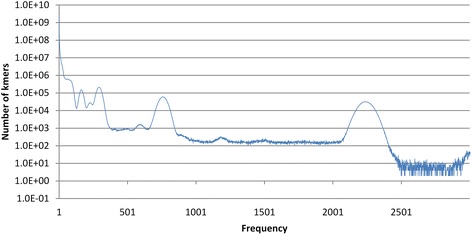
Frequency histogram of long k-mers computed from a simulated community genome. The histogram was generated by counting 20-mers from the synthetic dataset assembled from 500 distinct bacterial genomes, using 150 bp read length and an abundance distribution spanning 4 orders of magnitude (see Methods). k-mer frequencies are shown on the x axis; the number of distinct k-mers with a given frequency are shown on the y axis

To extract the coverage signal, reads are first decomposed into long k-mers (25-mers by default). Practically, each k-mer is paired with its reverse complement to account for the lack of knowledge of the strand the sequence is read from. The k-mer counting step is implemented using a large lock-free concurrent hash table. This data structure can be modified by multiple threads concurrently while reducing the lock contention that arise when several threads access the same synchronized hash map simultaneously. The improved scalability is achieved by using a finer grained strategy where locks are applied to segments instead of the whole map, enabling “lock-free” concurrent hash maps to behave close to the ideal, i.e., running *n* threads increases throughput *n* times. The output of the counting process can be viewed as a histogram or spectrum (see Fig. 1, which displays the logarithm of the number of distinct k-mers as a function of their coverage), which serves two roles. First, it is the input for the EM engine that will fit a mixture of Poisson distributions to it. Second, it enables a first pass filtering on the data, e.g., by removing unique or rare k-mers. In addition to dramatically reducing the dimensionality of the k-mer space and RAM memory requirements, this can filter out erroneous k-mers provoked by sequencing artefacts (deliberately injected at the dataset generation step to mimic real sequencing processes). Multiple worker threads analyze the content of reads in long k-mers and store their counts in a large hash-table supporting lock-free, concurrent modifications by multiple threads. Classes from the *java.util.concurrent* package (http://docs.oracle.com/javase/7/docs/api/java/util/concurrent/package-summary.html) were used in our implementation.

##### Expectation-Maximization (EM) step

We follow the same Expectation-Maximization approach as in [8] to identify the mixture of Poisson distributions that best fits the observed k-mer frequencies. Briefly, each Poisson distribution of parameter λ_i_ models the probability of certain k-mers coming from species (or group of species with similar abundance level) of relative abundance λ_i_. If S denotes the set of species, then the probability of k-mer k_j_ with n(k_j_) total counts coming from species (or group of species) S_i_ of relative abundance λ_i_ and genome length g_i_ (or total genome length in the case of a group of species of similar abundance) is given by [8]:

$$ P\left({k}_j\in {S}_i\left|n\left({k}_j\right)\right.\right)=\frac{{\mathsf{g}}_i}{{\displaystyle {\sum}_{m=1}^{\left|S\right|}{\mathsf{g}}_m}{\left(\frac{\lambda_m}{\lambda_i}\right)}^{n\left({k}_j\right)}e\left({\lambda}_i-{\lambda}_m\right)} $$

The EM process starts by defining n distributions with random initial parameters and iteratively updates parameters until convergence or until a maximum number of iterations is reached. The parameters of the Poisson distributions are updated iteratively according to:

$$ {\mathit{\mathsf{g}}}_i={\displaystyle \sum_{j=1}^KP\left({k}_j\in {S}_i\left|n\left({k}_j\right)\right.\right)} $$

$$ {\lambda}_i=\frac{{\displaystyle {\sum}_{j=1}^Kn\left({k}_j\right)P\left({k}_j\in {S}_i\left|n\left({k}_j\right)\right.\right)}}{{\mathit{\mathsf{g}}}_i} $$

##### Read assignment step

Both reads and clusters are represented as vectors in k-mer space, and the similarity of read r_m_ to cluster S_i_ is measured as the cosine similarity of their corresponding vectors. Each read r_m_ is represented as:

$$ \overrightarrow{r_m}=\left\langle n\left({k}_j,{r}_m\right)\right\rangle, j=1,\dots, \left|K\right| $$

Similarly, each abundance cluster is represented as:

$$ \overrightarrow{S_i}=\left\langle P\left({k}_j\in {S}_i\left|n\left({k}_j\right)\right.\right)\right\rangle, j=1,\dots, \left|K\right| $$

Read r_m_ is then assigned according to:

$$ S\left({r}_m\right)= argma{x}_i\left( cos\left(\overrightarrow{r_m},\;\overrightarrow{S_i}\right)\right)= argma{x}_i\left(\frac{{\displaystyle {\sum}_{j=1}^{\left|K\right|}{r}_{mj}{S}_{ij}}}{\sqrt{{\displaystyle {\sum}_{j=1}^{\left|K\right|}{r}_{mj}^2}}\sqrt{{\displaystyle {\sum}_{j=1}^{\left|K\right|}{S}_{ij}^2}}}\right) $$

Since the entries of both read and cluster vectors are non-negative, cosine similarities take value in the [0,1] interval.

#### Composition-based clustering

Our composition-based clustering implementation is compared to MetaCluster [14], a well recognized and state-of-the-art family of programs that share the same rationale. As different flavors of MetaCluster have been developed over time, differing in various pre and post-processing steps, we performed our comparisons against MetaCluster’s core algorithm, which is shared by all its versions and roughly corresponds to MetaCluster 3.0.

The compositional module geometrizes the reads by measuring their content in short k-mers (typically of length 4 or 5), and performs a k-means clustering of the sequences in k-mer space using Spearman’s footrule distance, which operates on rank vectors and is defined as: $F\left(\overrightarrow{r_m},\overrightarrow{S_i}\right)={\displaystyle {\sum}_{j=1}^{\left|K\right|}\left|{R}_j\left({r}_m\right)-{R}_j(Si)\right|}.$

Our k-means implementation uses multiple worker threads (one for each cluster prototype) to calculate in parallel the distances between read vectors and cluster centroids, while others re-compute centroids and assign reads to clusters under the supervision of a coordinating thread.

#### Chaining and two-level (2L) pipeline

The two clustering modules described in the previous sections are combined into a two-level clustering pipeline. The main idea of this integration is to smooth abundance variance by performing an initial coverage-based clustering; the resulting clusters, typically containing sequences from many distinct organisms of similar abundance, being then separately partitioned into finer grained composition-based clusters through independent executions of the compositional module. Alternatively, the more homogeneous sequence coverage achieved by execution of the first module can be leveraged by assembly engines, resulting in improved genome recovery (see section Use of the binning modules for *de novo* sequence “pre-assembly” and Table 3).

During execution of the integrated two-level pipeline, the number n_CB_ of output clusters for each instance of composition-based clustering can be automatically estimated from its parent abundance-based clustering. As is apparent from the EM update formulas, EM convergence yields both abundance estimate *λ*_*i*_ for each cluster together with its associated (meta)-genome length g_i_, which can be converted into a bin richness estimate, e.g., by assuming an average bacterial genome size.

Thus, the only parameter left to the user is the initial number n_AB_ of abundance classes, which can be rationalized in terms of the expected range of variation between cell numbers in the sample analyzed and the maximal (theoretical) resolution limit of the clustering algorithm. In our experience, we found that n_AB_ values ranging from 5 to 8 turned out to work well.

#### Inter-sample mapping

The integrated two-level pipeline thus performs a nested clustering at the intra-sample level. A third, composition-based, module is then used to relate two-level clusters across samples; this mapping leads to the definition of third-level (3L) clusters.

Practically, the third module operates in three steps: i) it computes a distance matrix between two-level clusters, ii) it then builds a hierarchical structure (dendrogram) from this distance matrix, iii) this dendrogram is cut, the resulting clades defining the final third-level clusters. We briefly expand on those steps.

##### Distance matrix computation

We chose the d_2_S statistic [12] as a dissimilarity measure to build a distance matrix from the two-level clusters, each of these being represented as a count vector for short (e.g., 4 to 7 mers) k-mers computed from their constituting reads.

The d_2_S dissimilarity between two count vectors is a centralized version of the well-known D_2_S measure, the latter simply being the dot product of the two vectors. For each k-mer count value, the centralization entails subtracting the expected count of the given k-mer under a given sequence background model. This genomic background is typically modeled using Markov models of various orders, though the cost associated with computing higher order Markov models frequently results in analyses being restricted to 0 order ones, as was done here. The d_2_S statistic takes values from 0 to 1, with 0, 0.5 and 1 corresponding to totally correlated, uncorrelated and anti-correlated pairs of sequences respectively.

##### Hierarchical clustering and dynamic tree cut

The d_2_S based distance matrix is then clustered using an agglomerative hierarchical method with an average linkage criterion to yield a tree of two-level clusters. Branches of the resulting tree are cut dynamically as described in [13], using default “shape” parameters to yield the final third-level (3L) clusters.

## Results and Discussion

### Performance of individual clustering modules and their chaining against state of the art

**Fig. 2:**
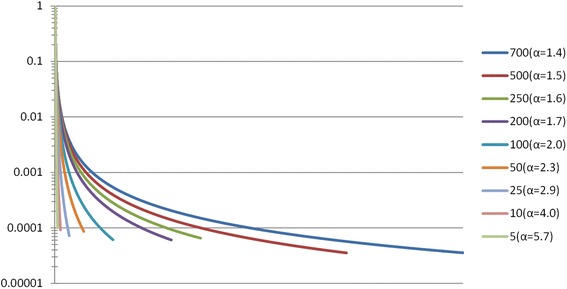
Abundance distributions of synthetic bacterial communities. Abundance distribution of sampled species for various datasets of increasing complexity, ranging from 5 to 700 distinct genomes (see main Text and Methods). A four order of magnitude difference between the number of cells from the most abundant *versus* less abundant organisms is used to determine power law parameters for each dataset (see Methods)

The accuracy of the first two clustering modules was extensively evaluated against state of the art software by benchmark experiments using synthetic bacterial communities assembled from real genomes, under empirical sequence error models and abundance distributions, and spanning a large range of species richness (see Methods and Fig. 2). The modules were evaluated both separately and integrated into a pipeline, without loss of accuracy. Figure 4 shows that the individual abundance and compositional modules have accuracies comparable to state of the art software, while runtime performance was dramatically improved due to parallelization and the use of large lock-free concurrent hash maps.

### Relevance to biomarker discovery use cases

**Fig. 3:**
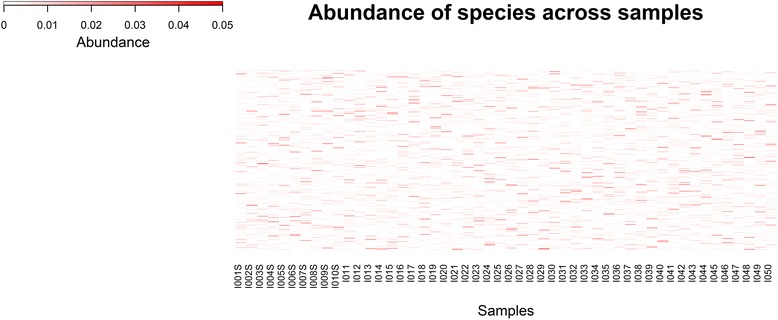
Heat map showing sampling levels for the 700 distinct genomes (rows) in each of the 50 samples (columns). Abundance levels of bacterial genomes across the 50 microbiome samples used to investigate the biomarker discovery use case

**Fig. 4:**
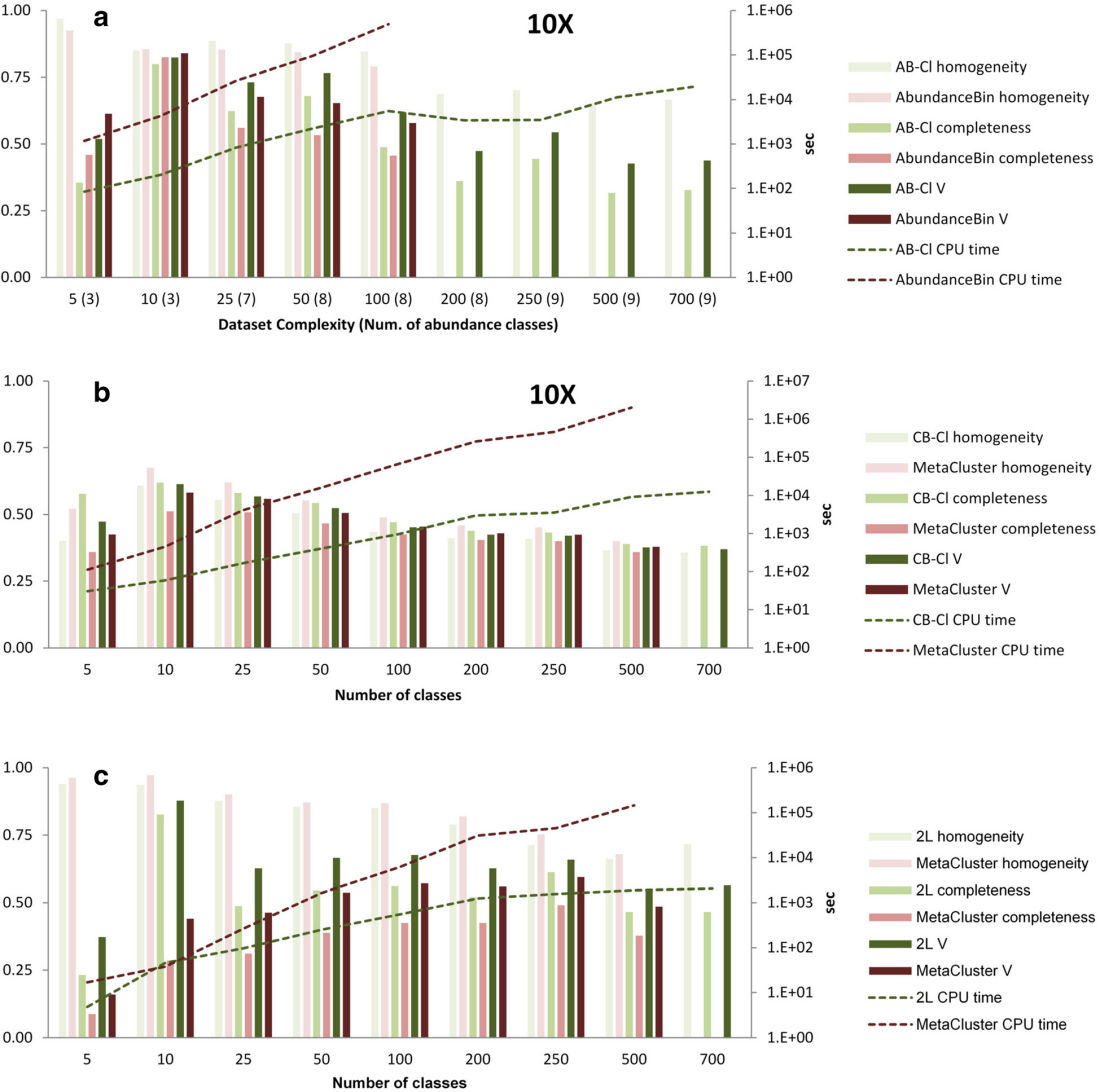
Evaluation of individual clustering modules and their chaining. **a** Comparison of the coverage-based clustering module (AB-Cl) *versus* AbundanceBin [8]. Groups of six bars for each sample of increasing complexity represent homogeneity, completeness and V1-measure (measured on the left axis) for AbundanceBin (*red bars*) and AB-Cl (*green bars*). Dotted lines denote execution time normalized per core (in logarithmic scale, on the right axis). Missing points result from AbundanceBin failing to process the datasets with 100 or more distinct genomes. **b** Comparison of the composition-based module (CB-Cl) *versus* MetaCluster [14]. Groups of six bars for each sample of increasing complexity represent homogeneity, completeness and V1-measure (measured on the left axis) for MetaCluster (*red bars*) and CB-Cl (*green bars*). Dotted lines denote execution time normalized per core (in logarithmic scale, on the right axis). Missing points result from MetaCluster failing to process the datasets with more than 500 distinct genomes. **c** Evaluation of the integrated two-level (2L) pipeline. The AB-Cl module was used for first level clustering, followed by either the CB-Cl module (*green*) or MetaCluster (*red*) for second level clustering. Dotted lines denote execution time normalized per core (in logarithmic scale, on the right axis). Missing points for the last dataset (700 distinct genomes) are due to the MetaCluster computation failing to complete

We assessed the usefulness of the binning scheme with respect to a simulated biomarker discovery use case consisting in the “de novo” identification of low abundance sequences correlated to disease status from metagenomic (e.g., microbiome) datasets derived from a cohort comprising healthy and sick individuals (see Methods and Fig. 3). The unassembled reads from 50 metagenomic datasets were processed by the three level binning pipeline, resulting in 6523 two-level (intra-sample) clusters, which in turn yielded 1664 third-level (cross-sample) clusters. Using the known reads to genome assignments as ground truth yielded homogeneity, completeness and V-measure of 0.61, 0.62 and 0.61 respectively, coherent with the evaluation metrics from the two-level pipeline (Fig. 4c).

We then evaluated the strength of the correlation between microbiome donor’s health status and the third-level (3L) clusters treated as variables by first binarizing the sample-by-3L-cluster matrix, marking cells SV_ij_ with 1 if 3L-cluster V_j_ contains sequences from sample S_i_, and leaving them marked with 0 otherwise. More refined binarization schemes will be explored in the future. The 3L-cluster variables were then ranked according to their mutual information with respect to the health status of microbiome hosts.

**Fig. 5:**
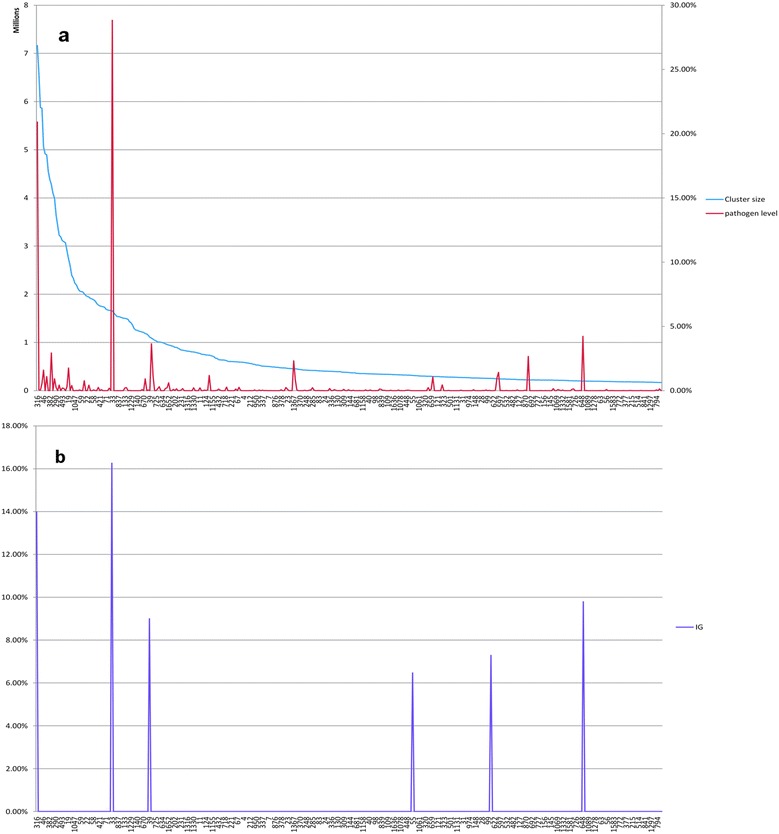
Evaluation of final third-level (3L) clusters. **a** Third-level clusters’ content in target (pathogen) genome sequences. 3L-clusters (labelled with their identifier on the x axis) are sorted according to their size (measured on the left axis, blue line). Red peaks show the fraction of total pathogen reads embedded in each 3L-cluster (measured on the right axis). **b** Relevance of final third-level clusters to disease status. 3L-clusters coordinates on the x axis are the same as in Fig. 5a; purple peaks represent information gain (IG) for each 3L-cluster with respect to sick *versus* healthy class assignments (see Methods). Note that only six 3L-clusters have IG values above background: the three highest red peaks (representing 3L-clusters embodying the bulk of the pathogen genome) correspond to the three highest IG purple peaks (first, second and last peaks); the remaining high IG peaks are correlated to the opposite (i.e., healthy) label, and are devoid of sequences from the pathogen strain

Remarkably, only six 3L-clusters displayed information gain values above background (Fig. 5b), with the three most highly correlated ones embodying the bulk of the reads sampled from the target (pathogen) genome (Fig. 5a and b). Aligning reads from the most highly correlated 3L-cluster to the target genome indicated that these sequences encompassed 66 % of its length; this proportion increased to 87 % when adding reads from the second most highly correlated cluster, after which the genomic coverage leveled off (Additional file 1: Table S1).

These results illustrate that the three-level binning pipeline can dramatically reduce (from 1664 to 6) the number of 3L-cluster variables in a biologically sensible manner, thereby providing valuable features to downstream supervised methods for predictive model development.

The results also indicate that the observed levels of cluster inhomogeneity are not impairing the discriminative value of the final 3L-clusters. In particular, the co-segregation of sequences from the strain closely related to the target genome did not disrupt the information gain based ranking, even when sequences from this strain were occurring at levels similar to those from the pathogen strain itself (note that the two strains share about 98 % nucleotide level identity). This observation is crucial, as the resolution limit of composition-based methods will inherently result in some level of noise, e.g., the separation of genomes from different strains is well beyond the power of composition-based methods alone. Moreover, pathogen-related clusters remained consistently ranked as the most highly correlated variables even when removing up to half of the pathogen containing microbiomes (i.e., keeping only five of these, data not shown). Thus, contrary to binning methods that rely on a relatively large number of samples in order to extract a sufficiently robust abundance covariation signal (e.g., about 20 samples in [15], 50 samples in [5], and 30 to 50 samples in [16]), the algorithm presented here is able to leverage a useful signal from a much smaller number of samples.

### Identification of sequence clusters from the *E. coli O104:H4* genome causative of the 2011 STEC outbreak in Germany from a cohort of 53 microbiomes

**Fig. 6:**
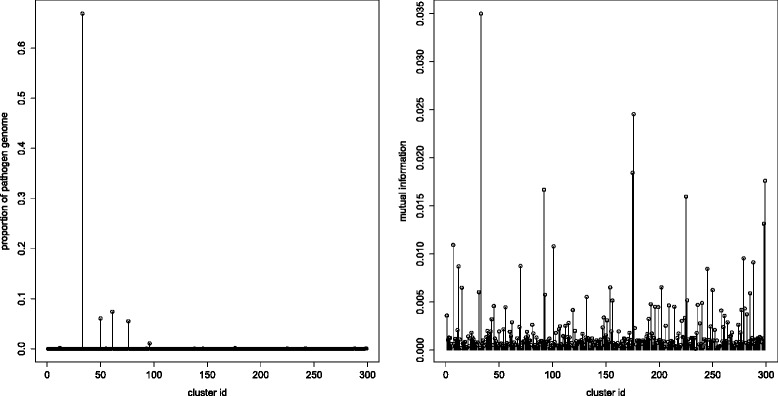
Evaluation of clusters of contigs from the STEC outbreak microbiomes (see main text). (*Left panel*) Clusters’ content in pathogen (*E. coli O104:H4*) genome sequence. Peaks show the fraction of the *E. coli O104:H4* genome embedded in each cluster computed by the CB-Cl module (k-mer size = 6, 300 output clusters (filtering out one dubious cluster containing more than 10 % of the original sequences); clusters are arbitrarily ordered on the x axis). (*Right panel*) Strength of the association between clusters and disease status. Cluster coordinates on the x axis are the same as in the left panel; peaks represent mutual information for each cluster with respect to the infection status of the microbiome donors (see main text). The cluster with the highest mutual information value encompasses about 70 % of the *E. coli O104:H4* genome sequence

In order to assess the ability of our clustering software to identify pathogen-derived sequences from real cohort data in an unsupervised way, we analyzed microbiome data generated during the 2011 STEC outbreak in Germany. Starting with the roughly 150,000 contigs resulting from a cross-assembly of the 53 microbiomes described in [5], we tested the ability of our compositional module to accurately cluster contigs and enable the reliable identification of clusters derived from the STEC causing strain by using meta-data pertaining to the health status of microbiome donors. In order to achieve this, we defined a binarization scheme for the cluster by sample matrix similar to the one described above for the analysis of 3L-clusters. This binarization entailed transforming the original contig by sample matrix (created by mapping the raw reads onto the contigs [5]) into a cluster by sample matrix, and was performed straightforwardly by registering a cluster in a sample if at least one of its component sequences (i.e., contigs) was present in the given sample, as recorded in the original abundance matrix [5]. The clusters from the resulting binarized matrix were then treated as variables and ranked according to their mutual information with respect to the infection status. The results of this experiment are summarized in Fig. 6, which makes apparent that the most highly correlated cluster (highest mutual information on the right panel of Fig. 6) covers almost 70 % of the *E. coli O104:H4* genome (left panel of Fig. 6). On the other hand, 72 % of the sequence content of this cluster is derived from the *E. coli O104:H4* genome, with more than 40 % of the remaining (i.e., not originating from the *E. coli O104:H4* strain) sequences still being assigned at the *E. coli* species level by Kraken [17], consistent with the segregation in the cohort of distinct *E. coli* strains that can not be resolved on the basis of a compositional signal alone. The left panel of this figure also shows that the bulk of the STEC genome is distributed among very few (four) clusters.

### Use of the binning modules for *de novo* sequence “pre-assembly”

Finally, in addition to the use cases described above, the binning modules have also been used for sequence pre-assembly in several metagenomic projects carried out in our institute and/or by collaborators. They were instrumental in reconstructing nearly complete genomes from all the members of a low complexity pyrene degrading enrichment culture (Table 2, and Adam I.K. et al., under review), and in recovering the complete genomes of key player bacteria in two anaerobic phenanthrene degrading enrichment cultures of medium complexity, i.e. harboring about 50 distinct genomes ([18] and Himmelberg A. et al., manuscript in preparation). Table 3 summarizes the results from one of these experiments, showing that the initial global metagenome assembly actually targeted only two (Bin IV and Bin V) out of the five abundance classes, while reads from the most abundant bin –whose estimated size is consistent with the occurrence within it of a single 6.5–6.8 Mbp genome- were discarded. Specifically assembling the reads from this bin (Bin I) allowed to recover a 6.5 Mbp genome in a single scaffold, which was estimated to be 99 % complete based on the distribution of 247 lineage specific single copy marker genes from [19].

**Table 2:** Reconstruction of individual genomes from a low-complexity pyrene degrading bacterial consortium using the coverage-based clustering module (AB-Cl) for sequence pre-assembly

Abundance class (Bin #)	Number of classified reads	Bin coverage estimate	% Binned reads mapped on reconstructed genome	% Total reads mapped on reconstructed genome	Genomes in given bin (% binned sequences mapped to it)
Bin I	168591494	1389	82 %	76,52 %	Bordetella (82 %)
Bin II	9445028	51	97 %	5,12 %	Mycobacterium (97 %)
Bin III	1870808	19	98 %	1,04 %	Stenotrophomonas (72 %), Sphingopyxis (22 %), Mycobacterium (3 %)
Bin IV	1331276	11	98 %	0,83 %	Sphingopyxis (76 %), Stenotrophomonas (19 %), Bordetella (3 %)

The completeness of the reconstructed genomes was assessed using lineage specific marker genes with CheckM [19], and yielded completeness estimates ranging from 97 to 99 %, with less than 2 % contamination and negligible strain level heterogeneity. Derived from Adam I.K. et al.*,* under review

**Table 3:** Coverage-based binning can enhance the recovery of individual genomes from metagenomes

Abundance class (Bin #)	% Binned reads in global assembly	% Binned reads from “key player”	Bin coverage (estimated by CB-Cl)	Bin coverage measured (from read mapping)	Bin size (estimated by CB-Cl)	Bin size measured (assembly length)	%Reads used in assembly of individual bins
Bin I	0.2 %	91.3 %	581	656	6.8 Mbp	6.5 Mbp	91.3 %
Bin II	0.5 %	1.3 %	317	306	7.0 Mbp	8.9 Mbp	86.3 %
Bin III	1.6 %	0.0 %	140	152	20.4 Mbp	21.2 Mbp	86.2 %
Bin IV	49.8 %	0.0 %	47	44	38.9 Mbp	37.6 Mbp	71.4 %
Bin V	37.7 %	0.0 %	14	17	117 Mbp	39 Mbp	30.4 %

Raw reads generated from a poly-aromatic hydrocarbon degrading enrichment culture [18] were assembled (with the ALLPATHS program [20]) globally on one hand, and segregated into 5 abundance classes (bins) followed by targeted assembly of reads from individual classes on the other hand. The assembly of reads with the highest coverage (Bin I) led to the reconstruction of a single 6.5 Mbp genome (key player), which is missing in the global metagenome assembly

## Conclusions

We have presented a set of sequence clustering modules and their application to biomarker (e.g., genomes of pathogenic strains) discovery from complex synthetic and real metagenomics datasets. Although initially designed for “assembly-free” analyses of single samples, we have demonstrated their relevance to experimental setups involving multiple samples through the use of the d_2_S “alignment-free statistic” to map clusters across samples, and have further illustrated how the binning modules can be used for *de novo* “pre-assembly” by segregating unassembled reads into biologically relevant partitions. By combining read-based with assembly-based analyses, this approach exemplifies the strategy advocated in [4] for moving towards genome-centric metagenomics. In the future, we intend to extend the presented methods in order to fully exploit the coverage covariation signal that is embedded in the larger cohorts that are increasingly becoming available for investigating the biomedical impacts of variation in the human microbiome.

## Additional files

Additional file 1: Contains two tables: the first table displays the effect on the coverage of the pathogenic genome of iteratively adding reads from the three most discriminative final (3 L) clusters; the second table lists the accession numbers of the 700 bacterial genomes used for generating the distinct simulated datasets. (DOC 576 kb) Additional file 2: Contains a figure illustrating the definition of reference abundance classes for evaluating the accuracy of the abundance-based clustering module. (DOC 88 kb)
