# Supplementary material for: A scalable assembly-free variable selection algorithm for biomarker discovery from metagenomes
Source: BMC Bioinformatics. 2016 Aug 19;17:311. doi: 10.1186/s12859-016-1186-3 (PMC4992282; doi:10.1186/s12859-016-1186-3)
Supplement: Additional file 1: — Contains two tables: the first table displays the effect on the coverage of the pathogenic genome of iteratively adding reads from the three most discriminative final (3 L) clusters; the second table lists the accession numbers of the 700 bacterial genomes used for generating the distinct simulated datasets. (DOC 576 kb) [file 12859_2016_1186_MOESM1_ESM.doc]

**Supplementary Table 1:**

| Cumulated reads from the three most discriminative 3L-clusters | Pathogen genome coverage |
| --- | --- |
| Reads from strongest peak (3L-cluster 161) | 65.8% |
| Reads from two strongest peaks (3L-clusters 161 and 135) | 86.5% |
| Reads from three strongest peaks (3L-clusters 161, 135 and 32) | 87.2% |

Coverage of the target (pathogen) strain’s genome by reads from the three final third-level (3L) clusters most highly correlated to disease status (see Methods and Figure 5).

**Supplementary Table 2:**

| |NC_000917.1| | Archaeoglobus fulgidus DSM 4304, complete genome |
| --- | --- |
| |NC_000964.3| | Bacillus subtilis subsp. subtilis str. 168 chromosome, complete genome |
| |NC_002570.2| | Bacillus halodurans C-125 chromosome, complete genome |
| |NC_002662.1| | Lactococcus lactis subsp. lactis Il1403 chromosome, complete genome |
| |NC_002663.1| | Pasteurella multocida subsp. multocida str. Pm70 chromosome, complete genome |
| |NC_002677.1| | Mycobacterium leprae TN chromosome, complete genome |
| |NC_002696.2| | Caulobacter crescentus CB15 chromosome, complete genome |
| |NC_002754.1| | Sulfolobus solfataricus P2 chromosome, complete genome |
| |NC_002929.2| | Bordetella pertussis Tohama I chromosome, complete genome |
| |NC_002932.3| | Chlorobium tepidum TLS chromosome, complete genome |
| |NC_002935.2| | Corynebacterium diphtheriae NCTC 13129 chromosome, complete genome |
| |NC_002939.5| | Geobacter sulfurreducens PCA chromosome, complete genome |
| |NC_002942.5| | Legionella pneumophila subsp. pneumophila str. Philadelphia 1 chromosome, complete genome |
| |NC_002946.2| | Neisseria gonorrhoeae FA 1090 chromosome, complete genome |
| |NC_002950.2| | Porphyromonas gingivalis W83 chromosome, complete genome |
| |NC_002952.2| | Staphylococcus aureus subsp. aureus MRSA252 chromosome, complete genome |
| |NC_002967.9| | Treponema denticola ATCC 35405 chromosome, complete genome |
| |NC_002973.6| | Listeria monocytogenes serotype 4b str. F2365 chromosome, complete genome |
| |NC_002977.6| | Methylococcus capsulatus str. Bath chromosome, complete genome |
| |NC_003028.3| | Streptococcus pneumoniae TIGR4 chromosome, complete genome |
| |NC_003098.1| | Streptococcus pneumoniae R6, complete genome |
| |NC_003106.2| | Sulfolobus tokodaii str. 7 chromosome, complete genome |
| |NC_003112.2| | Neisseria meningitidis MC58 chromosome, complete genome |
| |NC_003116.1| | Neisseria meningitidis Z2491 chromosome, complete genome |
| |NC_003210.1| | Listeria monocytogenes EGD-e, complete genome |
| |NC_003364.1| | Pyrobaculum aerophilum str. IM2 chromosome, complete genome |
| |NC_003450.3| | Corynebacterium glutamicum ATCC 13032, complete genome |
| |NC_003454.1| | Fusobacterium nucleatum subsp. nucleatum ATCC 25586 chromosome, complete genome |
| |NC_003869.1| | Thermoanaerobacter tengcongensis MB4 chromosome, complete genome |
| |NC_003901.1| | Methanosarcina mazei Go1 chromosome, complete genome |
| |NC_003923.1| | Staphylococcus aureus subsp. aureus MW2, complete genome |
| |NC_004113.1| | Thermosynechococcus elongatus BP-1 chromosome, complete genome |
| |NC_004116.1| | Streptococcus agalactiae 2603V/R chromosome, complete genome |
| |NC_004193.1| | Oceanobacillus iheyensis HTE831 chromosome, complete genome |
| |NC_004350.2| | Streptococcus mutans UA159 chromosome, complete genome |
| |NC_004368.1| | Streptococcus agalactiae NEM316, complete genome |
| |NC_004757.1| | Nitrosomonas europaea ATCC 19718 chromosome, complete genome |
| |NC_005070.1| | Synechococcus sp. WH 8102, complete genome |
| |NC_005071.1| | Prochlorococcus marinus str. MIT 9313 chromosome, complete genome |
| |NC_005090.1| | Wolinella succinogenes DSM 1740 chromosome, complete genome |
| |NC_005363.1| | Bdellovibrio bacteriovorus HD100, complete genome |
| |NC_005861.1| | Candidatus Protochlamydia amoebophila UWE25 chromosome, complete genome |
| |NC_005966.1| | Acinetobacter sp. ADP1 chromosome, complete genome |
| |NC_006085.1| | Propionibacterium acnes KPA171202 chromosome, complete genome |
| |NC_006087.1| | Leifsonia xyli subsp. xyli str. CTCB07 chromosome, complete genome |
| |NC_006177.1| | Symbiobacterium thermophilum IAM 14863 chromosome, complete genome |
| |NC_006270.3| | Bacillus licheniformis ATCC 14580 chromosome, complete genome |
| |NC_006300.1| | Mannheimia succiniciproducens MBEL55E chromosome, complete genome |
| |NC_006322.1| | Bacillus licheniformis DSM 13 = ATCC 14580 chromosome, complete genome |
| |NC_006512.1| | Idiomarina loihiensis L2TR chromosome, complete genome |
| |NC_006526.2| | Zymomonas mobilis subsp. mobilis ZM4 chromosome, complete genome |
| |NC_006576.1| | Synechococcus elongatus PCC 6301 chromosome, complete genome |
| |NC_006624.1| | Thermococcus kodakarensis KOD1 chromosome, complete genome |
| |NC_006958.1| | Corynebacterium glutamicum ATCC 13032, complete genome |
| |NC_007181.1| | Sulfolobus acidocaldarius DSM 639 chromosome, complete genome |
| |NC_007204.1| | Psychrobacter arcticus 273-4 chromosome, complete genome |
| |NC_007333.1| | Thermobifida fusca YX chromosome, complete genome |
| |NC_007404.1| | Thiobacillus denitrificans ATCC 25259 chromosome, complete genome |
| |NC_007406.1| | Nitrobacter winogradskyi Nb-255 chromosome, complete genome |
| |NC_007432.1| | Streptococcus agalactiae A909 chromosome, complete genome |
| |NC_007498.2| | Pelobacter carbinolicus DSM 2380 chromosome, complete genome |
| |NC_007503.1| | Carboxydothermus hydrogenoformans Z-2901 chromosome, complete genome |
| |NC_007512.1| | Chlorobium luteolum DSM 273 chromosome, complete genome |
| |NC_007513.1| | Synechococcus sp. CC9902 chromosome, complete genome |
| |NC_007514.1| | Chlorobium chlorochromatii CaD3 chromosome, complete genome |
| |NC_007516.1| | Synechococcus sp. CC9605, complete genome |
| |NC_007519.1| | Desulfovibrio alaskensis G20 chromosome, complete genome |
| |NC_007520.2| | Thiomicrospira crunogena XCL-2 chromosome, complete genome |
| |NC_007575.1| | Sulfurimonas denitrificans DSM 1251 chromosome, complete genome |
| |NC_007622.1| | Staphylococcus aureus RF122, complete genome |
| |NC_007644.1| | Moorella thermoacetica ATCC 39073 chromosome, complete genome |
| |NC_007722.1| | Erythrobacter litoralis HTCC2594 chromosome, complete genome |
| |NC_007759.1| | Syntrophus aciditrophicus SB chromosome, complete genome |
| |NC_007775.1| | Synechococcus sp. JA-3-3Ab chromosome, complete genome |
| |NC_007776.1| | Synechococcus sp. JA-2-3B'a(2-13) chromosome, complete genome |
| |NC_007795.1| | Staphylococcus aureus subsp. aureus NCTC 8325 chromosome, complete genome |
| |NC_007796.1| | Methanospirillum hungatei JF-1 chromosome, complete genome |
| |NC_007947.1| | Methylobacillus flagellatus KT, complete genome |
| |NC_007955.1| | Methanococcoides burtonii DSM 6242, complete genome |
| |NC_007963.1| | Chromohalobacter salexigens DSM 3043 chromosome, complete genome |
| |NC_008148.1| | Rubrobacter xylanophilus DSM 9941 chromosome, complete genome |
| |NC_008260.1| | Alcanivorax borkumensis SK2 chromosome, complete genome |
| |NC_008261.1| | Clostridium perfringens ATCC 13124 chromosome, complete genome |
| |NC_008319.1| | Synechococcus sp. CC9311, complete genome |
| |NC_008340.1| | Alkalilimnicola ehrlichii MLHE-1 chromosome, complete genome |
| |NC_008343.1| | Granulibacter bethesdensis CGDNIH1 chromosome, complete genome |
| |NC_008346.1| | Syntrophomonas wolfei subsp. wolfei str. Goettingen chromosome, complete genome |
| |NC_008347.1| | Maricaulis maris MCS10 chromosome, complete genome |
| |NC_008358.1| | Hyphomonas neptunium ATCC 15444 chromosome, complete genome |
| |NC_008533.1| | Streptococcus pneumoniae D39 chromosome, complete genome |
| |NC_008555.1| | Listeria welshimeri serovar 6b str. SLCC5334 chromosome, complete genome |
| |NC_008571.1| | Gramella forsetii KT0803 chromosome, complete genome |
| |NC_008578.1| | Acidothermus cellulolyticus 11B chromosome, complete genome |
| |NC_008593.1| | Clostridium novyi NT chromosome, complete genome |
| |NC_008618.1| | Bifidobacterium adolescentis ATCC 15703 chromosome, complete genome |
| |NC_008639.1| | Chlorobium phaeobacteroides DSM 266 chromosome, complete genome |
| |NC_008767.1| | Neisseria meningitidis FAM18 chromosome, complete genome |
| |NC_008789.1| | Halorhodospira halophila SL1 chromosome, complete genome |
| |NC_008820.1| | Prochlorococcus marinus str. MIT 9303 chromosome, complete genome |
| |NC_009004.1| | Lactococcus lactis subsp. cremoris MG1363 chromosome, complete genome |
| |NC_009009.1| | Streptococcus sanguinis SK36 chromosome, complete genome |
| |NC_009012.1| | Clostridium thermocellum ATCC 27405 chromosome, complete genome |
| |NC_009051.1| | Methanoculleus marisnigri JR1 chromosome, complete genome |
| |NC_009053.1| | Actinobacillus pleuropneumoniae serovar 5b str. L20 chromosome, complete genome |
| |NC_009138.1| | Herminiimonas arsenicoxydans chromosome, complete genome |
| |NC_009253.1| | Desulfotomaculum reducens MI-1 chromosome, complete genome |
| |NC_009376.1| | Pyrobaculum arsenaticum DSM 13514 chromosome, complete genome |
| |NC_009379.1| | Polynucleobacter necessarius subsp. asymbioticus QLW-P1DMWA-1 chromosome, complete genome |
| |NC_009437.1| | Caldicellulosiruptor saccharolyticus DSM 8903 chromosome, complete genome |
| |NC_009440.1| | Metallosphaera sedula DSM 5348 chromosome, complete genome |
| |NC_009442.1| | Streptococcus suis 05ZYH33 chromosome, complete genome |
| |NC_009443.1| | Streptococcus suis 98HAH33, complete genome |
| |NC_009454.1| | Pelotomaculum thermopropionicum SI chromosome, complete genome |
| |NC_009464.1| | Methanocella arvoryzae MRE50 chromosome, complete genome |
| |NC_009481.1| | Synechococcus sp. WH 7803 chromosome, complete genome |
| |NC_009482.1| | Synechococcus sp. RCC307 chromosome, complete genome |
| |NC_009488.1| | Orientia tsutsugamushi str. Boryong, complete genome |
| |NC_009494.2| | Legionella pneumophila str. Corby chromosome, complete genome |
| |NC_009613.3| | Flavobacterium psychrophilum JIP02/86 complete genome |
| |NC_009641.1| | Staphylococcus aureus subsp. aureus str. Newman chromosome, complete genome |
| |NC_009655.1| | Actinobacillus succinogenes 130Z chromosome, complete genome |
| |NC_009659.1| | Janthinobacterium sp. Marseille chromosome, complete genome |
| |NC_009663.1| | Sulfurovum sp. NBC37-1 chromosome, complete genome |
| |NC_009697.1| | Clostridium botulinum A str. ATCC 19397 chromosome, complete genome |
| |NC_009698.1| | Clostridium botulinum A str. Hall chromosome, complete genome |
| |NC_009712.1| | Methanoregula boonei 6A8 chromosome, complete genome |
| |NC_009719.1| | Parvibaculum lavamentivorans DS-1 chromosome, complete genome |
| |NC_009725.1| | Bacillus amyloliquefaciens FZB42, complete genome |
| |NC_009782.1| | Staphylococcus aureus subsp. aureus Mu3, complete genome |
| |NC_009785.1| | Streptococcus gordonii str. Challis substr. CH1 chromosome, complete genome |
| |NC_009828.1| | Thermotoga lettingae TMO chromosome, complete genome |
| |NC_009848.1| | Bacillus pumilus SAFR-032 chromosome, complete genome |
| |NC_009850.1| | Arcobacter butzleri RM4018 chromosome, complete genome |
| |NC_009922.1| | Alkaliphilus oremlandii OhILAs chromosome, complete genome |
| |NC_009943.1| | Desulfococcus oleovorans Hxd3 chromosome, complete genome |
| |NC_009954.1| | Caldivirga maquilingensis IC-167 chromosome, complete genome |
| |NC_010003.1| | Petrotoga mobilis SJ95 chromosome, complete genome |
| |NC_010080.1| | Lactobacillus helveticus DPC 4571, complete genome |
| |NC_010120.1| | Neisseria meningitidis 053442 chromosome, complete genome |
| |NC_010168.1| | Renibacterium salmoninarum ATCC 33209 chromosome, complete genome |
| |NC_010278.1| | Actinobacillus pleuropneumoniae serovar 3 str. JL03 chromosome, complete genome |
| |NC_010320.1| | Thermoanaerobacter sp. X514 chromosome, complete genome |
| |NC_010321.1| | Thermoanaerobacter pseudethanolicus ATCC 33223 chromosome, complete genome |
| |NC_010337.2| | Heliobacterium modesticaldum Ice1 chromosome, complete genome |
| |NC_010380.1| | Streptococcus pneumoniae Hungary19A-6, complete genome |
| |NC_010424.1| | Candidatus Desulforudis audaxviator MP104C chromosome, complete genome |
| |NC_010513.1| | Xylella fastidiosa M12 chromosome, complete genome |
| |NC_010519.1| | Haemophilus somnus 2336 chromosome, complete genome |
| |NC_010545.1| | Corynebacterium urealyticum DSM 7109 chromosome, complete genome |
| |NC_010582.1| | Streptococcus pneumoniae CGSP14 chromosome, complete genome |
| |NC_010609.1| | Lactobacillus reuteri JCM 1112, complete genome |
| |NC_010610.1| | Lactobacillus fermentum IFO 3956, complete genome |
| |NC_010617.1| | Kocuria rhizophila DC2201, complete genome |
| |NC_010645.1| | Bordetella avium 197N chromosome, complete genome |
| |NC_010655.1| | Akkermansia muciniphila ATCC BAA-835 chromosome, complete genome |
| |NC_010723.1| | Clostridium botulinum E3 str. Alaska E43 chromosome, complete genome |
| |NC_010729.1| | Porphyromonas gingivalis ATCC 33277, complete genome |
| |NC_010794.1| | Methylacidiphilum infernorum V4, complete genome |
| |NC_010803.1| | Chlorobium limicola DSM 245 chromosome, complete genome |
| |NC_010831.1| | Chlorobium phaeobacteroides BS1 chromosome, complete genome |
| |NC_010999.1| | Lactobacillus casei BL23 chromosome, complete genome |
| |NC_011026.1| | Chloroherpeton thalassium ATCC 35110 chromosome, complete genome |
| |NC_011027.1| | Chlorobaculum parvum NCIB 8327 chromosome, complete genome |
| |NC_011060.1| | Pelodictyon phaeoclathratiforme BU-1 chromosome, complete genome |
| |NC_011072.1| | Streptococcus pneumoniae G54 chromosome, complete genome |
| |NC_011206.1| | Acidithiobacillus ferrooxidans ATCC 53993 chromosome, complete genome |
| |NC_011386.1| | Oligotropha carboxidovorans OM5 chromosome, complete genome |
| |NC_011567.1| | Anoxybacillus flavithermus WK1 chromosome, complete genome |
| |NC_011593.1| | Bifidobacterium longum subsp. infantis ATCC 15697 chromosome, complete genome |
| |NC_011595.1| | Acinetobacter baumannii AB307-0294, complete genome |
| |NC_011660.1| | Listeria monocytogenes HCC23 chromosome, complete genome |
| |NC_011761.1| | Acidithiobacillus ferrooxidans ATCC 23270 chromosome, complete genome |
| |NC_011769.1| | Desulfovibrio vulgaris str. 'Miyazaki F' chromosome, complete genome |
| |NC_011832.1| | Methanosphaerula palustris E1-9c chromosome, complete genome |
| |NC_011852.1| | Haemophilus parasuis SH0165 chromosome, complete genome |
| |NC_011883.1| | Desulfovibrio desulfuricans subsp. desulfuricans str. ATCC 27774 chromosome, complete genome |
| |NC_011896.1| | Mycobacterium leprae Br4923 chromosome, complete genome |
| |NC_011898.1| | Clostridium cellulolyticum H10 chromosome, complete genome |
| |NC_011899.1| | Halothermothrix orenii H 168 chromosome, complete genome |
| |NC_011900.1| | Streptococcus pneumoniae ATCC 700669, complete genome |
| |NC_011901.1| | Thioalkalivibrio sulfidophilus HL-EbGr7 chromosome, complete genome |
| |NC_011916.1| | Caulobacter crescentus NA1000 chromosome, complete genome |
| |NC_011992.1| | Acidovorax ebreus TPSY chromosome, complete genome |
| |NC_012121.1| | Staphylococcus carnosus subsp. carnosus TM300 chromosome, complete genome |
| |NC_012466.1| | Streptococcus pneumoniae JJA, complete genome |
| |NC_012467.1| | Streptococcus pneumoniae P1031, complete genome |
| |NC_012468.1| | Streptococcus pneumoniae 70585, complete genome |
| |NC_012469.1| | Streptococcus pneumoniae Taiwan19F-14 chromosome, complete genome |
| |NC_012470.1| | Streptococcus equi subsp. zooepidemicus, complete genome |
| |NC_012471.1| | Streptococcus equi subsp. equi 4047, complete genome |
| |NC_012483.1| | Acidobacterium capsulatum ATCC 51196 chromosome, complete genome |
| |NC_012488.1| | Listeria monocytogenes Clip81459, complete genome |
| |NC_012559.1| | Laribacter hongkongensis HLHK9, complete genome |
| |NC_012563.1| | Clostridium botulinum A2 str. Kyoto chromosome, complete genome |
| |NC_012588.1| | Sulfolobus islandicus M.14.25 chromosome, complete genome |
| |NC_012589.1| | Sulfolobus islandicus L.S.2.15 chromosome, complete genome |
| |NC_012622.1| | Sulfolobus islandicus Y.G.57.14 chromosome, complete genome |
| |NC_012632.1| | Sulfolobus islandicus M.16.27 chromosome, complete genome |
| |NC_012673.1| | Exiguobacterium sp. AT1b chromosome, complete genome |
| |NC_012691.1| | Tolumonas auensis DSM 9187 chromosome, complete genome |
| |NC_012695.1| | Burkholderia pseudomallei MSHR346 chromosome I, complete sequence |
| |NC_012704.1| | Corynebacterium kroppenstedtii DSM 44385 chromosome, complete genome |
| |NC_012726.1| | Sulfolobus islandicus M.16.4 chromosome, complete genome |
| |NC_012779.2| | Edwardsiella ictaluri 93-146 chromosome, complete genome |
| |NC_012781.1| | Eubacterium rectale ATCC 33656, complete genome |
| |NC_012785.1| | Kosmotoga olearia TBF 19.5.1, complete genome |
| |NC_012803.1| | Micrococcus luteus NCTC 2665 chromosome, complete genome |
| |NC_012804.1| | Thermococcus gammatolerans EJ3 chromosome, complete genome |
| |NC_012891.1| | Streptococcus dysgalactiae subsp. equisimilis GGS_124 chromosome 1, complete sequence |
| |NC_012913.1| | Aggregatibacter aphrophilus NJ8700 chromosome, complete genome |
| |NC_012924.1| | Streptococcus suis SC84, complete genome |
| |NC_012968.1| | Methylotenera mobilis JLW8 chromosome, complete genome |
| |NC_012984.1| | Lactobacillus plantarum JDM1, complete genome |
| |NC_013016.1| | Neisseria meningitidis alpha14 chromosome, complete genome |
| |NC_013062.1| | Flavobacteriaceae bacterium 3519-10, complete genome |
| |NC_013124.1| | Acidimicrobium ferrooxidans DSM 10331 chromosome, complete genome |
| |NC_013158.1| | Halorhabdus utahensis DSM 12940 chromosome, complete genome |
| |NC_013162.1| | Capnocytophaga ochracea DSM 7271 chromosome, complete genome |
| |NC_013165.1| | Slackia heliotrinireducens DSM 20476 chromosome, complete genome |
| |NC_013166.1| | Kangiella koreensis DSM 16069 chromosome, complete genome |
| |NC_013169.1| | Kytococcus sedentarius DSM 20547 chromosome, complete genome |
| |NC_013172.1| | Brachybacterium faecium DSM 4810 chromosome, complete genome |
| |NC_013173.1| | Desulfomicrobium baculatum DSM 4028, complete genome |
| |NC_013174.1| | Jonesia denitrificans DSM 20603 chromosome, complete genome |
| |NC_013192.1| | Leptotrichia buccalis C-1013-b chromosome, complete genome |
| |NC_013198.1| | Lactobacillus rhamnosus GG chromosome, complete genome |
| |NC_013204.1| | Eggerthella lenta DSM 2243 chromosome, complete genome |
| |NC_013222.1| | Robiginitalea biformata HTCC2501 chromosome, complete genome |
| |NC_013260.1| | Candidatus Methylomirabilis oxyfera, complete genome |
| |NC_013315.1| | Clostridium difficile CD196 chromosome, complete genome |
| |NC_013316.1| | Clostridium difficile R20291 chromosome, complete genome |
| |NC_013410.1| | Fibrobacter succinogenes subsp. succinogenes S85 chromosome, complete genome |
| |NC_013422.1| | Halothiobacillus neapolitanus c2 chromosome, complete genome |
| |NC_013512.1| | Sulfurospirillum deleyianum DSM 6946 chromosome, complete genome |
| |NC_013520.1| | Veillonella parvula DSM 2008 chromosome, complete genome |
| |NC_013665.1| | Methanocella paludicola SANAE chromosome, complete genome |
| |NC_013714.1| | Bifidobacterium dentium Bd1 chromosome, complete genome |
| |NC_013715.1| | Rothia mucilaginosa DY-18 chromosome, complete genome |
| |NC_013740.1| | Acidaminococcus fermentans DSM 20731 chromosome, complete genome |
| |NC_013768.1| | Listeria monocytogenes 08-5923, complete genome |
| |NC_013790.1| | Methanobrevibacter ruminantium M1 chromosome, complete genome |
| |NC_013798.1| | Streptococcus gallolyticus UCN34 chromosome, complete genome |
| |NC_013849.1| | Ferroglobus placidus DSM 10642 chromosome, complete genome |
| |NC_013853.1| | Streptococcus mitis B6, complete genome |
| |NC_013891.1| | Listeria seeligeri serovar 1/2b str. SLCC3954 chromosome, complete genome |
| |NC_013892.1| | Xenorhabdus bovienii SS-2004 chromosome, complete genome |
| |NC_013893.1| | Staphylococcus lugdunensis HKU09-01 chromosome, complete genome |
| |NC_013921.1| | Thermoanaerobacter italicus Ab9 chromosome, complete genome |
| |NC_013943.1| | Denitrovibrio acetiphilus DSM 12809 chromosome, complete genome |
| |NC_013946.1| | Meiothermus ruber DSM 1279 chromosome, complete genome |
| |NC_013959.1| | Sideroxydans lithotrophicus ES-1 chromosome, complete genome |
| |NC_013974.1| | Clostridium difficile BI9 chromosome |
| |NC_014008.1| | Coraliomargarita akajimensis DSM 45221 chromosome, complete genome |
| |NC_014010.1| | Candidatus Puniceispirillum marinum IMCC1322 chromosome, complete genome |
| |NC_014033.1| | Prevotella ruminicola 23 chromosome, complete genome |
| |NC_014039.1| | Propionibacterium acnes SK137 chromosome, complete genome |
| |NC_014098.1| | Kyrpidia tusciae DSM 2912 chromosome, complete genome |
| |NC_014106.1| | Lactobacillus crispatus ST1, complete genome |
| |NC_014125.1| | Legionella pneumophila 2300/99 Alcoy chromosome, complete genome |
| |NC_014150.1| | Brachyspira murdochii DSM 12563 chromosome, complete genome |
| |NC_014151.1| | Cellulomonas flavigena DSM 20109 chromosome, complete genome |
| |NC_014152.1| | Thermincola potens JR chromosome, complete genome |
| |NC_014165.1| | Thermobispora bispora DSM 43833 chromosome, complete genome |
| |NC_014166.1| | Arcobacter nitrofigilis DSM 7299 chromosome, complete genome |
| |NC_014168.1| | Segniliparus rotundus DSM 44985 chromosome, complete genome |
| |NC_014169.1| | Bifidobacterium longum subsp. longum JDM301 chromosome, complete genome |
| |NC_014206.1| | Geobacillus sp. C56-T3 chromosome, complete genome |
| |NC_014207.1| | Methylotenera versatilis 301 chromosome, complete genome |
| |NC_014209.1| | Thermoanaerobacter mathranii subsp. mathranii str. A3 chromosome, complete genome |
| |NC_014215.1| | Propionibacterium freudenreichii subsp. shermanii CIRM-BIA1 chromosome, complete genome |
| |NC_014216.1| | Desulfurivibrio alkaliphilus AHT2 chromosome, complete genome |
| |NC_014219.1| | Bacillus selenitireducens MLS10 chromosome, complete genome |
| |NC_014220.1| | Syntrophothermus lipocalidus DSM 12680 chromosome, complete genome |
| |NC_014221.1| | Truepera radiovictrix DSM 17093 chromosome, complete genome |
| |NC_014230.1| | Croceibacter atlanticus HTCC2559 chromosome, complete genome |
| |NC_014246.1| | Mobiluncus curtisii ATCC 43063 chromosome, complete genome |
| |NC_014251.1| | Streptococcus pneumoniae TCH8431/19A chromosome, complete genome |
| |NC_014259.1| | Acinetobacter oleivorans DR1 chromosome, complete genome |
| |NC_014307.1| | Ralstonia solanacearum CFBP2957 chromosome, complete genome |
| |NC_014313.1| | Hyphomicrobium denitrificans ATCC 51888 chromosome, complete genome |
| |NC_014329.1| | Corynebacterium pseudotuberculosis FRC41 chromosome, complete genome |
| |NC_014330.1| | Brachyspira pilosicoli 95/1000 chromosome, complete genome |
| |NC_014363.1| | Olsenella uli DSM 7084 chromosome, complete genome |
| |NC_014365.1| | Desulfarculus baarsii DSM 2075 chromosome, complete genome |
| |NC_014375.1| | Brevundimonas subvibrioides ATCC 15264 chromosome, complete genome |
| |NC_014377.1| | Thermosediminibacter oceani DSM 16646 chromosome, complete genome |
| |NC_014378.1| | Acetohalobium arabaticum DSM 5501 chromosome, complete genome |
| |NC_014392.1| | Caldicellulosiruptor obsidiansis OB47 chromosome, complete genome |
| |NC_014394.1| | Gallionella capsiferriformans ES-2 chromosome, complete genome |
| |NC_014410.1| | Thermoanaerobacterium thermosaccharolyticum DSM 571 chromosome, complete genome |
| |NC_014414.1| | Parvularcula bermudensis HTCC2503 chromosome, complete genome |
| |NC_014472.1| | Maribacter sp. HTCC2170 chromosome, complete genome |
| |NC_014479.1| | Bacillus subtilis subsp. spizizenii str. W23 chromosome, complete genome |
| |NC_014484.1| | Spirochaeta thermophila DSM 6192 chromosome, complete genome |
| |NC_014494.1| | Streptococcus pneumoniae AP200 chromosome, complete genome |
| |NC_014498.1| | Streptococcus pneumoniae 670-6B chromosome, complete genome |
| |NC_014506.1| | Sulfurimonas autotrophica DSM 16294 chromosome, complete genome |
| |NC_014507.1| | Methanoplanus petrolearius DSM 11571 chromosome, complete genome |
| |NC_014532.1| | Halomonas elongata DSM 2581 chromosome, complete genome |
| |NC_014537.1| | Vulcanisaeta distributa DSM 14429 chromosome, complete genome |
| |NC_014538.1| | Thermoanaerobacter sp. X513 chromosome, complete genome |
| |NC_014551.1| | Bacillus amyloliquefaciens DSM 7, complete genome |
| |NC_014614.1| | [Clostridium] sticklandii, complete genome |
| |NC_014616.1| | Bifidobacterium bifidum S17 chromosome, complete genome |
| |NC_014638.1| | Bifidobacterium bifidum PRL2010 chromosome, complete genome |
| |NC_014639.1| | Bacillus atrophaeus 1942 chromosome, complete genome |
| |NC_014643.1| | Rothia dentocariosa ATCC 17931 chromosome, complete genome |
| |NC_014652.1| | Caldicellulosiruptor hydrothermalis 108 chromosome, complete genome |
| |NC_014654.1| | Halanaerobium hydrogeniformans chromosome, complete genome |
| |NC_014655.1| | Leadbetterella byssophila DSM 17132 chromosome, complete genome |
| |NC_014656.1| | Bifidobacterium longum subsp. longum BBMN68 chromosome, complete genome |
| |NC_014657.1| | Caldicellulosiruptor owensensis OL chromosome, complete genome |
| |NC_014664.1| | Rhodomicrobium vannielii ATCC 17100 chromosome, complete genome |
| |NC_014720.1| | Caldicellulosiruptor kronotskyensis 2002 chromosome, complete genome |
| |NC_014733.1| | Methylovorus sp. MP688 chromosome, complete genome |
| |NC_014734.1| | Paludibacter propionicigenes WB4 chromosome, complete genome |
| |NC_014738.1| | Riemerella anatipestifer ATCC 11845 = DSM 15868 chromosome, complete genome |
| |NC_014752.1| | Neisseria lactamica 020-06 chromosome, complete genome |
| |NC_014820.1| | Cenarchaeum symbiosum A, complete genome |
| |NC_014828.1| | Ethanoligenens harbinense YUAN-3 chromosome, complete genome |
| |NC_014830.1| | Intrasporangium calvum DSM 43043 chromosome, complete genome |
| |NC_014831.1| | Thermaerobacter marianensis DSM 12885 chromosome, complete genome |
| |NC_014836.1| | Desulfurispirillum indicum S5 chromosome, complete genome |
| |NC_014844.1| | Desulfovibrio aespoeensis Aspo-2 chromosome, complete genome |
| |NC_014924.1| | Pseudoxanthomonas suwonensis 11-1 chromosome, complete genome |
| |NC_014925.1| | Staphylococcus pseudintermedius HKU10-03 chromosome, complete genome |
| |NC_014933.1| | Bacteroides helcogenes P 36-108 chromosome, complete genome |
| |NC_014935.1| | Nitratifractor salsuginis DSM 16511 chromosome, complete genome |
| |NC_014958.1| | Deinococcus maricopensis DSM 21211 chromosome, complete genome |
| |NC_014960.1| | Anaerolinea thermophila UNI-1, complete genome |
| |NC_014964.1| | Thermoanaerobacter brockii subsp. finnii Ako-1 chromosome, complete genome |
| |NC_014972.1| | Desulfobulbus propionicus DSM 2032 chromosome, complete genome |
| |NC_014976.1| | Bacillus subtilis BSn5 chromosome, complete genome |
| |NC_015067.1| | Bifidobacterium longum subsp. longum JCM 1217 chromosome, complete genome |
| |NC_015125.1| | Microbacterium testaceum StLB037, complete genome |
| |NC_015144.1| | Weeksella virosa DSM 16922 chromosome, complete genome |
| |NC_015151.1| | Vulcanisaeta moutnovskia 768-28 chromosome, complete genome |
| |NC_015152.1| | Spirochaeta sp. Buddy chromosome, complete genome |
| |NC_015167.1| | Cellulophaga lytica DSM 7489 chromosome, complete genome |
| |NC_015172.1| | Syntrophobotulus glycolicus DSM 8271 chromosome, complete genome |
| |NC_015216.1| | Methanobacterium sp. AL-21 chromosome, complete genome |
| |NC_015278.1| | Aerococcus urinae ACS-120-V-Col10a chromosome, complete genome |
| |NC_015311.1| | Prevotella denticola F0289 chromosome, complete genome |
| |NC_015387.1| | Marinithermus hydrothermalis DSM 14884 chromosome, complete genome |
| |NC_015388.1| | Desulfobacca acetoxidans DSM 11109 chromosome, complete genome |
| |NC_015389.1| | Coriobacterium glomerans PW2 chromosome, complete genome |
| |NC_015433.1| | Streptococcus suis ST3 chromosome, complete genome |
| |NC_015436.1| | Spirochaeta coccoides DSM 17374 chromosome, complete genome |
| |NC_015437.1| | Selenomonas sputigena ATCC 35185 chromosome, complete genome |
| |NC_015496.1| | Krokinobacter sp. 4H-3-7-5 chromosome, complete genome |
| |NC_015500.1| | Treponema brennaborense DSM 12168 chromosome, complete genome |
| |NC_015501.1| | Porphyromonas asaccharolytica DSM 20707 chromosome, complete genome |
| |NC_015518.1| | Acidianus hospitalis W1 chromosome, complete genome |
| |NC_015519.1| | Tepidanaerobacter sp. Re1 chromosome, complete genome |
| |NC_015520.1| | Mahella australiensis 50-1 BON chromosome, complete genome |
| |NC_015555.1| | Thermoanaerobacterium xylanolyticum LX-11 chromosome, complete genome |
| |NC_015558.1| | Streptococcus parauberis KCTC 11537 chromosome, complete genome |
| |NC_015559.1| | Marinomonas posidonica IVIA-Po-181 chromosome, complete genome |
| |NC_015565.1| | Desulfotomaculum carboxydivorans CO-1-SRB chromosome, complete genome |
| |NC_015571.1| | Porphyromonas gingivalis TDC60, complete genome |
| |NC_015573.1| | Desulfotomaculum kuznetsovii DSM 6115 chromosome, complete genome |
| |NC_015574.1| | Methanobacterium sp. SWAN-1 chromosome, complete genome |
| |NC_015577.1| | Treponema azotonutricium ZAS-9 chromosome, complete genome |
| |NC_015578.1| | Treponema primitia ZAS-2 chromosome, complete genome |
| |NC_015588.1| | Isoptericola variabilis 225 chromosome, complete genome |
| |NC_015589.1| | Desulfotomaculum ruminis DSM 2154 chromosome, complete genome |
| |NC_015600.1| | Streptococcus pasteurianus ATCC 43144, complete genome |
| |NC_015634.1| | Bacillus coagulans 2-6 chromosome, complete genome |
| |NC_015638.1| | Lacinutrix sp. 5H-3-7-4 chromosome, complete genome |
| |NC_015671.1| | Cellvibrio gilvus ATCC 13127 chromosome, complete genome |
| |NC_015672.1| | Flexistipes sinusarabici DSM 4947 chromosome, complete genome |
| |NC_015673.1| | Corynebacterium resistens DSM 45100 chromosome, complete genome |
| |NC_015676.1| | Methanosalsum zhilinae DSM 4017 chromosome, complete genome |
| |NC_015677.1| | Ramlibacter tataouinensis TTB310 chromosome, complete genome |
| |NC_015678.1| | Streptococcus parasanguinis ATCC 15912 chromosome, complete genome |
| |NC_015681.1| | Thermodesulfatator indicus DSM 15286 chromosome, complete genome |
| |NC_015683.1| | Corynebacterium ulcerans BR-AD22 chromosome, complete genome |
| |NC_015696.1| | Francisella sp. TX077308 chromosome, complete genome |
| |NC_015702.1| | Parachlamydia acanthamoebae UV-7 chromosome, complete genome |
| |NC_015707.1| | Thermotoga thermarum DSM 5069 chromosome, complete genome |
| |NC_015731.1| | Nitrosomonas sp. Is79A3 chromosome, complete genome |
| |NC_015732.1| | Spirochaeta caldaria DSM 7334 chromosome, complete genome |
| |NC_015737.1| | Clostridium sp. SY8519, complete genome |
| |NC_015738.1| | Eggerthella sp. YY7918, complete genome |
| |NC_015757.1| | Sulfobacillus acidophilus TPY chromosome, complete genome |
| |NC_015760.1| | Streptococcus salivarius CCHSS3, complete genome |
| |NC_015846.1| | Capnocytophaga canimorsus Cc5 chromosome, complete genome |
| |NC_015859.1| | Corynebacterium variabile DSM 44702 chromosome, complete genome |
| |NC_015873.1| | Megasphaera elsdenii DSM 20460, complete genome |
| |NC_015942.1| | Acidithiobacillus ferrivorans SS3 chromosome, complete genome |
| |NC_015945.1| | Muricauda ruestringensis DSM 13258 chromosome, complete genome |
| |NC_015949.1| | Caldicellulosiruptor lactoaceticus 6A chromosome, complete genome |
| |NC_015958.1| | Thermoanaerobacter wiegelii Rt8.B1 chromosome, complete genome |
| |NC_015964.1| | Haemophilus parainfluenzae T3T1, complete genome |
| |NC_015975.1| | Lactobacillus ruminis ATCC 27782 chromosome, complete genome |
| |NC_015977.1| | Roseburia hominis A2-183 chromosome, complete genome |
| |NC_016011.1| | Listeria ivanovii subsp. ivanovii PAM 55, complete genome |
| |NC_016023.1| | Bacillus coagulans 36D1 chromosome, complete genome |
| |NC_016026.1| | Micavibrio aeruginosavorus ARL-13 chromosome, complete genome |
| |NC_016041.1| | Glaciecola nitratireducens FR1064 chromosome, complete genome |
| |NC_016047.1| | Bacillus subtilis subsp. spizizenii TU-B-10 chromosome, complete genome |
| |NC_016051.1| | Thermococcus sp. AM4 chromosome, complete genome |
| |NC_016052.1| | Tetragenococcus halophilus NBRC 12172, complete genome |
| |NC_016077.1| | Acidaminococcus intestini RyC-MR95 chromosome, complete genome |
| |NC_016147.2| | Pseudoxanthomonas spadix BD-a59 chromosome, complete genome |
| |NC_016510.2| | Flavobacterium columnare ATCC 49512 chromosome, complete genome |
| |NC_016511.1| | Propionibacterium acnes TypeIA2 P.acn31 chromosome, complete genome |
| |NC_016512.1| | Propionibacterium acnes TypeIA2 P.acn17 chromosome, complete genome |
| |NC_016513.1| | Aggregatibacter actinomycetemcomitans ANH9381 chromosome, complete genome |
| |NC_016516.1| | Propionibacterium acnes TypeIA2 P.acn33 chromosome, complete genome |
| |NC_016593.1| | Geobacillus thermoleovorans CCB_US3_UF5 chromosome, complete genome |
| |NC_016599.1| | Owenweeksia hongkongensis DSM 17368 chromosome, complete genome |
| |NC_016603.1| | Acinetobacter calcoaceticus PHEA-2 chromosome, complete genome |
| |NC_016610.1| | Tannerella forsythia ATCC 43037 chromosome, complete genome |
| |NC_016616.1| | Dechlorosoma suillum PS chromosome, complete genome |
| |NC_016629.1| | Desulfovibrio africanus str. Walvis Bay chromosome, complete genome |
| |NC_016633.1| | Sphaerochaeta pleomorpha str. Grapes chromosome, complete genome |
| |NC_016645.1| | Pyrobaculum sp. 1860 chromosome, complete genome |
| |NC_016781.1| | Corynebacterium pseudotuberculosis 3/99-5 chromosome, complete genome |
| |NC_016782.1| | Corynebacterium diphtheriae 241 chromosome, complete genome |
| |NC_016783.1| | Corynebacterium diphtheriae INCA 402 chromosome, complete genome |
| |NC_016784.1| | Bacillus amyloliquefaciens subsp. plantarum CAU B946, complete genome |
| |NC_016785.1| | Corynebacterium diphtheriae CDCE 8392 chromosome, complete genome |
| |NC_016786.1| | Corynebacterium diphtheriae HC01 chromosome, complete genome |
| |NC_016787.1| | Corynebacterium diphtheriae HC03 chromosome, complete genome |
| |NC_016788.1| | Corynebacterium diphtheriae HC04 chromosome, complete genome |
| |NC_016789.1| | Corynebacterium diphtheriae PW8 chromosome, complete genome |
| |NC_016790.1| | Corynebacterium diphtheriae VA01 chromosome, complete genome |
| |NC_016799.1| | Corynebacterium diphtheriae 31A chromosome, complete genome |
| |NC_016800.1| | Corynebacterium diphtheriae BH8 chromosome, complete genome |
| |NC_016801.1| | Corynebacterium diphtheriae C7 (beta) chromosome, complete genome |
| |NC_016802.1| | Corynebacterium diphtheriae HC02 chromosome, complete genome |
| |NC_016803.1| | Desulfovibrio desulfuricans ND132 chromosome, complete genome |
| |NC_016808.1| | Pasteurella multocida 36950 chromosome, complete genome |
| |NC_016811.1| | Legionella pneumophila subsp. pneumophila ATCC 43290 chromosome, complete genome |
| |NC_016894.1| | Acetobacterium woodii DSM 1030 chromosome, complete genome |
| |NC_016912.1| | Staphylococcus aureus subsp. aureus VC40 chromosome, complete genome |
| |NC_016928.1| | Staphylococcus aureus subsp. aureus M013 chromosome, complete genome |
| |NC_016932.1| | Corynebacterium pseudotuberculosis 316 chromosome, complete genome |
| |NC_017025.1| | Flavobacterium indicum GPTSA100-9, complete genome |
| |NC_017031.1| | Corynebacterium pseudotuberculosis P54B96 chromosome, complete genome |
| |NC_017033.1| | Frateuria aurantia DSM 6220 chromosome, complete genome |
| |NC_017034.1| | Methanocella conradii HZ254 chromosome, complete genome |
| |NC_017038.1| | Synechocystis sp. PCC 6803 substr. GT-I, complete genome |
| |NC_017039.1| | Synechocystis sp. PCC 6803 substr. PCC-P, complete genome |
| |NC_017045.1| | Riemerella anatipestifer ATCC 11845 = DSM 15868 chromosome, complete genome |
| |NC_017052.1| | Synechocystis sp. PCC 6803 substr. PCC-N, complete genome |
| |NC_017059.1| | Rhodospirillum photometricum DSM 122, complete genome |
| |NC_017067.1| | Marinobacter hydrocarbonoclasticus ATCC 49840, complete genome |
| |NC_017094.1| | Leptospirillum ferrooxidans C2-3, complete genome |
| |NC_017095.1| | Fervidobacterium pennivorans DSM 9078 chromosome, complete genome |
| |NC_017098.1| | Spirochaeta africana DSM 8902 chromosome, complete genome |
| |NC_017167.1| | Alicyclobacillus acidocaldarius subsp. acidocaldarius Tc-4-1 chromosome, complete genome |
| |NC_017173.1| | Clostridium difficile CF5, complete genome |
| |NC_017174.1| | Clostridium difficile M120, complete genome |
| |NC_017178.1| | Clostridium difficile 2007855, complete genome |
| |NC_017187.1| | Arcobacter butzleri ED-1, complete genome |
| |NC_017188.1| | Bacillus amyloliquefaciens TA208 chromosome, complete genome |
| |NC_017191.1| | Bacillus amyloliquefaciens XH7 chromosome, complete genome |
| |NC_017195.1| | Bacillus subtilis subsp. subtilis str. RO-NN-1 chromosome, complete genome |
| |NC_017218.1| | Bifidobacterium breve ACS-071-V-Sch8b chromosome, complete genome |
| |NC_017219.1| | Bifidobacterium longum subsp. infantis ATCC 15697, complete genome |
| |NC_017223.1| | Bordetella pertussis CS chromosome, complete genome |
| |NC_017274.1| | Sulfolobus solfataricus 98/2 chromosome, complete genome |
| |NC_017275.1| | Sulfolobus islandicus HVE10/4 chromosome, complete genome |
| |NC_017276.1| | Sulfolobus islandicus REY15A chromosome, complete genome |
| |NC_017277.1| | Synechocystis sp. PCC 6803, complete genome |
| |NC_017299.1| | Clostridium botulinum H04402 065, complete genome |
| |NC_017300.1| | Corynebacterium pseudotuberculosis 1002 chromosome, complete genome |
| |NC_017301.1| | Corynebacterium pseudotuberculosis C231 chromosome, complete genome |
| |NC_017303.1| | Corynebacterium pseudotuberculosis I19 chromosome, complete genome |
| |NC_017304.1| | Clostridium thermocellum DSM 1313 chromosome, complete genome |
| |NC_017305.1| | Corynebacterium pseudotuberculosis PAT10 chromosome, complete genome |
| |NC_017306.1| | Corynebacterium pseudotuberculosis 42/02-A chromosome, complete genome |
| |NC_017307.1| | Corynebacterium pseudotuberculosis CIP 52.97 chromosome, complete genome |
| |NC_017308.1| | Corynebacterium pseudotuberculosis 1/06-A chromosome, complete genome |
| |NC_017316.1| | Enterococcus faecalis OG1RF chromosome, complete genome |
| |NC_017317.1| | Corynebacterium ulcerans 809 chromosome, complete genome |
| |NC_017337.1| | Staphylococcus aureus subsp. aureus ED133 chromosome, complete genome |
| |NC_017340.1| | Staphylococcus aureus 04-02981 chromosome, complete genome |
| |NC_017341.1| | Staphylococcus aureus subsp. aureus str. JKD6008 chromosome, complete genome |
| |NC_017347.1| | Staphylococcus aureus subsp. aureus T0131 chromosome, complete genome |
| |NC_017353.1| | Staphylococcus lugdunensis N920143, complete genome |
| |NC_017448.1| | Fibrobacter succinogenes subsp. succinogenes S85 chromosome, complete genome |
| |NC_017454.1| | Geobacter sulfurreducens KN400 chromosome, complete genome |
| |NC_017455.1| | Halanaerobium praevalens DSM 2228 chromosome, complete genome |
| |NC_017462.1| | Corynebacterium pseudotuberculosis 267 chromosome, complete genome |
| |NC_017464.1| | Ignavibacterium album JCM 16511 chromosome, complete genome |
| |NC_017465.1| | Lactobacillus fermentum CECT 5716 chromosome, complete genome |
| |NC_017482.1| | Lactobacillus rhamnosus GG, complete genome |
| |NC_017491.1| | Lactobacillus rhamnosus ATCC 8530 chromosome, complete genome |
| |NC_017501.1| | Neisseria meningitidis 8013, complete genome |
| |NC_017505.1| | Neisseria meningitidis alpha710 chromosome, complete genome |
| |NC_017512.1| | Neisseria meningitidis WUE 2594, complete genome |
| |NC_017513.1| | Neisseria meningitidis G2136 chromosome, complete genome |
| |NC_017514.1| | Neisseria meningitidis M01-240149 chromosome, complete genome |
| |NC_017515.1| | Neisseria meningitidis M04-240196 chromosome, complete genome |
| |NC_017516.1| | Neisseria meningitidis H44/76 chromosome, complete genome |
| |NC_017517.1| | Neisseria meningitidis M01-240355 chromosome, complete genome |
| |NC_017518.1| | Neisseria meningitidis NZ-05/33 chromosome, complete genome |
| |NC_017525.1| | Legionella pneumophila subsp. pneumophila str. Hextuple_2q chromosome, complete genome |
| |NC_017526.1| | Legionella pneumophila subsp. pneumophila str. Hextuple_3a chromosome, complete genome |
| |NC_017529.1| | Listeria monocytogenes L99, complete genome |
| |NC_017534.1| | Propionibacterium acnes 266 chromosome, complete genome |
| |NC_017535.1| | Propionibacterium acnes 6609 chromosome, complete genome |
| |NC_017537.1| | Listeria monocytogenes M7 chromosome, complete genome |
| |NC_017544.1| | Listeria monocytogenes 10403S chromosome, complete genome |
| |NC_017545.1| | Listeria monocytogenes J0161 chromosome, complete genome |
| |NC_017546.1| | Listeria monocytogenes FSL R2-561 chromosome, complete genome |
| |NC_017547.1| | Listeria monocytogenes Finland 1998 chromosome, complete genome |
| |NC_017550.1| | Propionibacterium acnes ATCC 11828 chromosome, complete genome |
| |NC_017567.1| | Streptococcus dysgalactiae subsp. equisimilis ATCC 12394 chromosome, complete genome |
| |NC_017568.1| | Staphylococcus pseudintermedius ED99 chromosome, complete genome |
| |NC_017569.1| | Riemerella anatipestifer RA-GD chromosome, complete genome |
| |NC_017576.1| | Streptococcus gallolyticus subsp. gallolyticus ATCC 43143, complete genome |
| |NC_017582.1| | Streptococcus equi subsp. zooepidemicus ATCC 35246 chromosome, complete genome |
| |NC_017583.1| | Spirochaeta thermophila DSM 6578 chromosome, complete genome |
| |NC_017591.1| | Streptococcus pneumoniae INV104, complete genome |
| |NC_017592.1| | Streptococcus pneumoniae OXC141, complete genome |
| |NC_017593.1| | Streptococcus pneumoniae INV200, complete genome |
| |NC_017595.1| | Streptococcus salivarius JIM8777, complete genome |
| |NC_017617.1| | Streptococcus suis GZ1 chromosome, complete genome |
| |NC_017618.1| | Streptococcus suis JS14 chromosome, complete genome |
| |NC_017619.1| | Streptococcus suis SS12 chromosome, complete genome |
| |NC_017620.1| | Streptococcus suis D9 chromosome, complete genome |
| |NC_017621.1| | Streptococcus suis D12 chromosome, complete genome |
| |NC_017622.1| | Streptococcus suis A7 chromosome, complete genome |
| |NC_017673.1| | Staphylococcus aureus subsp. aureus 71193 chromosome, complete genome |
| |NC_017728.1| | Listeria monocytogenes 07PF0776 chromosome, complete genome |
| |NC_017730.1| | Corynebacterium pseudotuberculosis 31 chromosome, complete genome |
| |NC_017743.1| | Bacillus sp. JS chromosome, complete genome |
| |NC_017763.1| | Staphylococcus aureus subsp. aureus HO 5096 0412, complete genome |
| |NC_017764.1| | Pasteurella multocida subsp. multocida str. 3480 chromosome, complete genome |
| |NC_017769.1| | Streptococcus pneumoniae ST556 chromosome, complete genome |
| |NC_017846.1| | Aggregatibacter actinomycetemcomitans D7S-1 chromosome, complete genome |
| |NC_017857.1| | Methylophaga sp. JAM1 chromosome, complete genome |
| |NC_017905.1| | Streptococcus parasanguinis FW213 chromosome, complete genome |
| |NC_017910.1| | Escherichia blattae DSM 4481 chromosome, complete genome |
| |NC_017945.1| | Corynebacterium pseudotuberculosis 258 chromosome, complete genome |
| |NC_017949.1| | Lactococcus lactis subsp. cremoris NZ9000 chromosome, complete genome |
| |NC_017950.1| | Streptococcus suis ST1 chromosome, complete genome |
| |NC_017999.1| | Bifidobacterium bifidum BGN4 chromosome, complete genome |
| |NC_018002.1| | Sulfurospirillum barnesii SES-3 chromosome, complete genome |
| |NC_018010.1| | Belliella baltica DSM 15883 chromosome, complete genome |
| |NC_018011.1| | Alistipes finegoldii DSM 17242 chromosome, complete genome |
| |NC_018013.1| | Aequorivita sublithincola DSM 14238 chromosome, complete genome |
| |NC_018016.1| | Ornithobacterium rhinotracheale DSM 15997 chromosome, complete genome |
| |NC_018019.1| | Corynebacterium pseudotuberculosis Cp162 chromosome, complete genome |
| |NC_018024.1| | Anaerobaculum mobile DSM 13181 chromosome, complete genome |
| |NC_018089.1| | Streptococcus mutans GS-5 chromosome, complete genome |
| |NC_018101.1| | Corynebacterium ulcerans 0102, complete genome |
| |NC_018140.1| | Legionella pneumophila subsp. pneumophila, complete genome |
| |NC_018142.1| | Propionibacterium propionicum F0230a chromosome, complete genome |
| |NC_018177.1| | Pseudomonas stutzeri DSM 10701 chromosome, complete genome |
| |NC_018178.1| | Melioribacter roseus P3M chromosome, complete genome |
| |NC_018227.1| | Methanoculleus bourgensis MS2, complete genome |
| |NC_018268.1| | Marinobacter sp. BSs20148 chromosome, complete genome |
| |NC_018485.1| | Methylocystis sp. SC2, complete genome |
| |NC_018518.1| | Bordetella pertussis 18323, complete genome |
| |NC_018520.1| | Bacillus subtilis QB928 chromosome, complete genome |
| |NC_018528.1| | Lactobacillus helveticus R0052 chromosome, complete genome |
| |NC_018584.1| | Listeria monocytogenes ATCC 19117, complete genome |
| |NC_018585.1| | Listeria monocytogenes SLCC2378, complete genome |
| |NC_018586.1| | Listeria monocytogenes SLCC2540, complete genome |
| |NC_018589.1| | Listeria monocytogenes SLCC2479, complete genome |
| |NC_018590.1| | Listeria monocytogenes SLCC2376, complete genome |
| |NC_018592.1| | Listeria monocytogenes SLCC5850, complete genome |
| |NC_018593.1| | Listeria monocytogenes SLCC7179, complete genome |
| |NC_018594.1| | Streptococcus pneumoniae SPNA45, complete genome |
| |NC_018604.1| | Brachyspira pilosicoli WesB complete genome |
| |NC_018607.1| | Brachyspira pilosicoli B2904 chromosome, complete genome |
| |NC_018608.1| | Staphylococcus aureus 08BA02176 chromosome, complete genome |
| |NC_018609.1| | Riemerella anatipestifer RA-CH-1 chromosome, complete genome |
| |NC_018630.1| | Streptococcus pneumoniae gamPNI0373 chromosome, complete genome |
| |NC_018642.1| | Listeria monocytogenes L312, complete genome |
| |NC_018646.1| | Streptococcus agalactiae GD201008-001 chromosome, complete genome |
| |NC_018649.1| | Leptospirillum ferriphilum ML-04 chromosome, complete genome |
| |NC_018665.1| | Exiguobacterium antarcticum B7 chromosome, complete genome |
| |NC_018690.1| | Actinobacillus suis H91-0380 chromosome, complete genome |
| |NC_018697.1| | Cycloclasticus sp. P1 chromosome, complete genome |
| |NC_018704.1| | Amphibacillus xylanus NBRC 15112, complete genome |
| |NC_018706.1| | Acinetobacter baumannii TYTH-1 chromosome, complete genome |
| |NC_018707.1| | Propionibacterium acnes C1 chromosome, complete genome |
| |NC_018712.1| | Streptococcus dysgalactiae subsp. equisimilis RE378, complete genome |
| |NC_018719.1| | Candidatus Nitrososphaera gargensis Ga9.2 chromosome, complete genome |
| |NC_018720.1| | Bifidobacterium asteroides PRL2011 chromosome, complete genome |
| |NC_018866.1| | Dehalobacter sp. DCA chromosome, complete genome |
| |NC_018867.1| | Dehalobacter sp. CF chromosome, complete genome |
| |NC_018870.1| | Thermacetogenium phaeum DSM 12270 chromosome, complete genome |
| |NC_018876.1| | Methanolobus psychrophilus R15 chromosome, complete genome |
| |NC_019042.1| | Streptococcus dysgalactiae subsp. equisimilis AC-2713, complete genome |
| |NC_019395.1| | Propionibacterium acidipropionici ATCC 4875 chromosome, complete genome |
| |NC_019425.2| | Carnobacterium maltaromaticum LMA28 complete genome |
| |NC_019556.1| | Listeria monocytogenes serotype 4b str. LL195, complete genome |
| |NC_019567.1| | Bdellovibrio bacteriovorus str. Tiberius chromosome, complete genome |
| |NC_019675.1| | Cyanobium gracile PCC 6307 chromosome, complete genome |
| |NC_019770.1| | Enterococcus faecalis str. Symbioflor 1, complete genome |
| |NC_019778.1| | Cyanobacterium stanieri PCC 7202 chromosome, complete genome |
| |NC_019779.1| | Halothece sp. PCC 7418 chromosome, complete genome |
| |NC_019780.1| | Dactylococcopsis salina PCC 8305 chromosome, complete genome |
| |NC_019792.1| | Natronobacterium gregoryi SP2 chromosome, complete genome |
| |NC_019842.1| | Bacillus amyloliquefaciens subsp. plantarum AS43.3 chromosome, complete genome |
| |NC_019896.1| | Bacillus subtilis subsp. subtilis str. BSP1 chromosome, complete genome |
| |NC_019902.1| | Thioalkalivibrio nitratireducens DSM 14787, complete genome |
| |NC_019903.1| | Desulfitobacterium dichloroeliminans LMG P-21439 chromosome, complete genome |
| |NC_019908.1| | Brachyspira pilosicoli P43/6/78 chromosome, complete genome |
| |NC_019943.1| | Methanoregula formicicum SMSP, complete genome |
| |NC_019954.1| | Tepidanaerobacter acetatoxydans Re1 complete genome |
| |NC_019964.1| | Halovivax ruber XH-70, complete genome |
| |NC_019978.1| | Halobacteroides halobius DSM 5150, complete genome |
| |NC_020125.1| | Riemerella anatipestifer RA-CH-2, complete genome |
| |NC_020134.1| | Clostridium stercorarium subsp. stercorarium DSM 8532, complete genome |
| |NC_020156.1| | Nonlabens dokdonensis DSW-6, complete genome |
| |NC_020210.1| | Geobacillus sp. GHH01, complete genome |
| |NC_020229.1| | Lactobacillus plantarum ZJ316, complete genome |
| |NC_020230.1| | Corynebacterium urealyticum DSM 7111, complete genome |
| |NC_020244.1| | Bacillus subtilis XF-1, complete genome |
| |NC_020246.1| | Sulfolobus acidocaldarius N8, complete genome |
| |NC_020247.1| | Sulfolobus acidocaldarius Ron12/I, complete genome |
| |NC_020388.1| | Natronomonas moolapensis 8.8.11 complete genome |
| |NC_020389.1| | Methanosarcina mazei Tuc01, complete genome |
| |NC_020409.1| | Desulfovibrio piezophilus C1TLV30, complete genome |
| |NC_020410.1| | Bacillus amyloliquefaciens subsp. plantarum UCMB5036 complete genome |
| |NC_020417.1| | Beta proteobacterium CB, complete genome |
| |NC_020418.1| | Morganella morganii subsp. morganii KT, complete genome |
| |NC_020449.1| | Candidatus Cloacamonas acidaminovorans str. Evry provisional genome sequence from WWE1 candidate division |
| |NC_020450.1| | Lactococcus lactis subsp. lactis IO-1 DNA, complete genome |
| |NC_020515.1| | Bibersteinia trehalosi USDA-ARS-USMARC-192, complete genome |
| |NC_020517.1| | Bifidobacterium breve UCC2003, complete genome |
| |NC_020518.1| | Escherichia coli str. K-12 substr. MDS42 DNA, complete genome |
| |NC_020519.1| | Corynebacterium glutamicum K051 complete genome, strain ATCC 13032, sub-strain K051 |
| |NC_020526.1| | Streptococcus suis SC070731, complete genome |
| |NC_020541.1| | Rhodanobacter sp. 2APBS1, complete genome |
| |NC_020546.1| | Bifidobacterium thermophilum RBL67, complete genome |
| |NC_020555.1| | Helicobacter cinaedi ATCC BAA-847 DNA, complete genome |
| |NC_020557.1| | Listeria monocytogenes La111 complete genome |
| |NC_020558.1| | Listeria monocytogenes N53-1 complete genome |
| |NC_020796.1| | Edwardsiella tarda C07-087, complete genome |
| |NC_020802.1| | Psychromonas sp. CNPT3, complete genome |
| |NC_020812.1| | Micavibrio aeruginosavorus EPB, complete genome |
| |NC_020813.1| | Bdellovibrio exovorus JSS, complete genome |
| |NC_020830.1| | Polaribacter sp. MED152, complete genome |
| |NC_020832.1| | Bacillus subtilis subsp. subtilis str. BAB-1, complete genome |
| |NC_020833.1| | Mannheimia haemolytica USDA-ARS-USMARC-183, complete genome |
| |NC_020834.1| | Mannheimia haemolytica USDA-ARS-SAM-185, complete genome |
| |NC_020887.1| | Clostridium stercorarium subsp. stercorarium DSM 8532, complete genome |
| |NC_020888.1| | Thalassolituus oleivorans MIL-1 complete genome |
| |NC_020891.1| | Clavibacter michiganensis subsp. nebraskensis NCPPB 2581 complete genome |
| |NC_020995.1| | Enterococcus casseliflavus EC20, complete genome |
| |NC_021003.1| | Streptococcus pneumoniae SPN032672 draft genome |
| |NC_021004.1| | Streptococcus pneumoniae SPN033038 draft genome |
| |NC_021005.1| | Streptococcus pneumoniae SPN994039 draft genome |
| |NC_021006.1| | Streptococcus pneumoniae SPN034156 draft genome |
| |NC_021008.1| | Bifidobacterium longum subsp. longum F8 draft genome |
| |NC_021009.1| | Coprococcus catus GD/7 draft genome |
| |NC_021010.1| | Eubacterium rectale DSM 17629 draft genome |
| |NC_021011.1| | Eubacterium siraeum 70/3 draft genome |
| |NC_021013.1| | Ruminococcus bromii L2-63 draft genome |
| |NC_021014.1| | Ruminococcus sp. SR1/5 draft genome |
| |NC_021015.1| | Ruminococcus torques L2-14 draft genome |
| |NC_021016.1| | Butyrate-producing bacterium SSC/2, complete genome |
| |NC_021018.1| | Coprococcus sp. ART55/1 draft genome |
| |NC_021020.1| | Faecalibacterium prausnitzii SL3/3 draft genome |
| |NC_021021.1| | Gordonibacter pamelaeae 7-10-1-b draft genome |
| |NC_021022.1| | Ruminococcus obeum A2-162 draft genome |
| |NC_021023.1| | Enterococcus sp. 7L76 draft genome |
| |NC_021024.1| | Butyrate-producing bacterium SM4/1, complete genome |
| |NC_021026.1| | Streptococcus pneumoniae SPN994038 draft genome |
| |NC_021028.1| | Streptococcus pneumoniae SPN034183 draft genome |
| |NC_021030.1| | Alistipes shahii WAL 8301 draft genome |
| |NC_021031.1| | Butyrivibrio fibrisolvens 16/4 draft genome |
| |NC_021035.1| | Butyrate-producing bacterium SS3/4, complete genome |
| |NC_021038.1| | Synergistetes bacterium SGP1, complete genome |
| |NC_021039.1| | Ruminococcus champanellensis 18P13, complete genome |
| |NC_021040.1| | Roseburia intestinalis M50/1 draft genome |
| |NC_021041.1| | Megamonas hypermegale ART12/1 draft genome |
| |NC_021042.1| | Faecalibacterium prausnitzii L2-6, complete genome |
| |NC_021043.1| | Eubacterium siraeum V10Sc8a draft genome |
| |NC_021044.1| | Eubacterium rectale M104/1 draft genome |
| |NC_021047.1| | Clostridium cf. saccharolyticum K10, complete genome |
| |NC_021058.1| | Sulfolobus islandicus LAL14/1, complete genome |
| |NC_021064.1| | Propionibacterium avidum 44067, complete genome |
| |NC_021081.1| | Meiothermus ruber DSM 1279 genome |
| |NC_021082.1| | Mannheimia haemolytica M42548, complete genome |
| |NC_021169.1| | Archaeoglobus sulfaticallidus PM70-1, complete genome |
| |NC_021172.1| | Hyphomicrobium denitrificans 1NES1, complete genome |
| |NC_021175.1| | Streptococcus oligofermentans AS 1.3089, complete genome |
| |NC_021213.1| | Streptococcus suis TL13, complete genome |
| |NC_021235.1| | Lactobacillus fermentum F-6, complete genome |
| |NC_021286.1| | Idiomarina loihiensis GSL 199, complete genome |
| |NC_021313.1| | Salinarchaeum sp. Harcht-Bsk1, complete genome |
| |NC_021314.1| | Streptococcus iniae SF1, complete genome |
| |NC_021350.1| | Legionella pneumophila subsp. pneumophila str. Thunder Bay, complete genome |
| |NC_021351.1| | Corynebacterium glutamicum SCgG1, complete genome |
| |NC_021352.1| | Corynebacterium glutamicum SCgG2, complete genome |
| |NC_021485.1| | Streptococcus agalactiae 09mas018883 complete genome |
| |NC_021486.1| | Streptococcus agalactiae ILRI005 complete genome |
| |NC_021487.1| | Chthonomonas calidirosea T49 complete genome |
| |NC_021507.1| | Streptococcus agalactiae ILRI112 complete genome |
| |NC_021521.1| | Haemophilus parasuis ZJ0906, complete genome |

Listing of the 700 genomes used in the experiments.
