# Supplementary material for: A scalable assembly-free variable selection algorithm for biomarker discovery from metagenomes
Source: BMC Bioinformatics. 2016 Aug 19;17:311. doi: 10.1186/s12859-016-1186-3 (PMC4992282; doi:10.1186/s12859-016-1186-3)
Supplement: Additional file 2: — Contains a figure illustrating the definition of reference abundance classes for evaluating the accuracy of the abundance-based clustering module. (DOC 88 kb) [file 12859_2016_1186_MOESM2_ESM.doc]

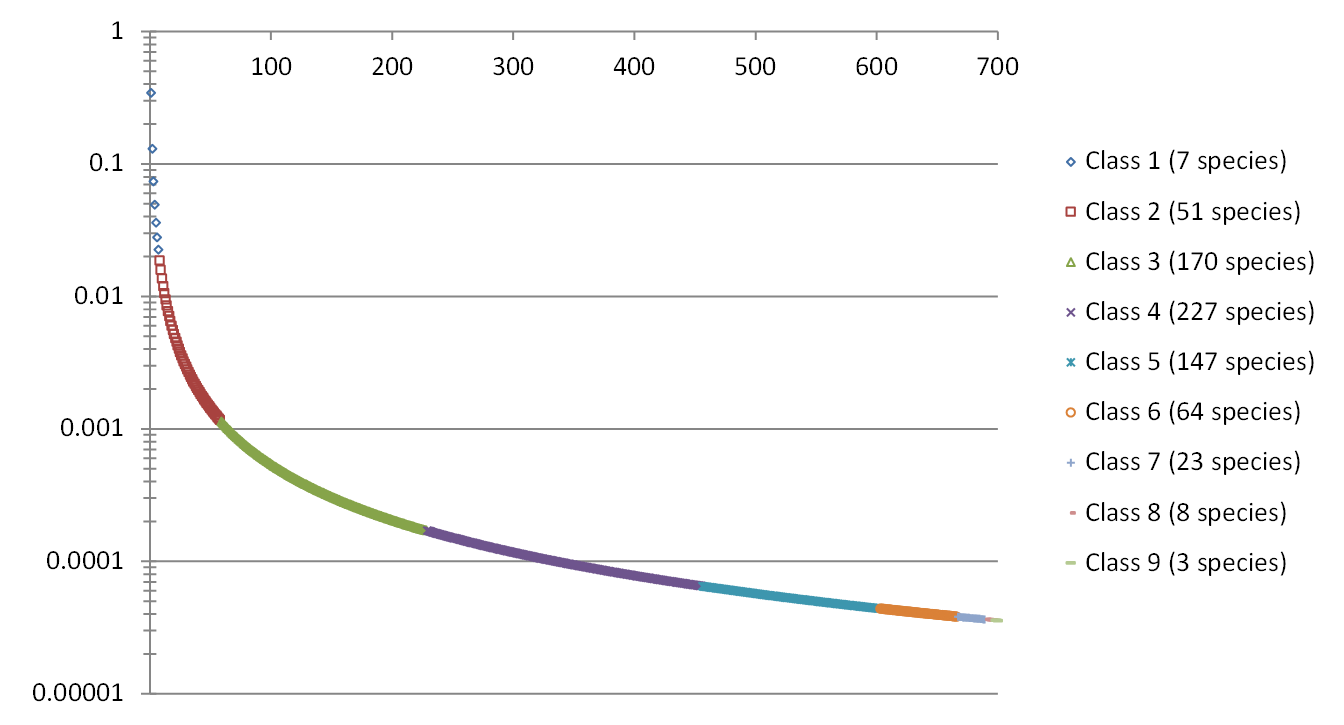


Supplementary Figure 1

**Definition of reference abundance classes for clustering evaluation purposes.**

Example of abundance classes defined from the 700 bacteria community genome. Organisms of similar abundance are grouped in classes in a way such that the cumulative relative abundance of a given class is at least twice that of its adjacent less abundant class (see Methods).
